# Understanding the role of the paramedic in primary care: a realist review

**DOI:** 10.1186/s12916-021-02019-z

**Published:** 2021-06-25

**Authors:** Georgette Eaton, Geoff Wong, Stephanie Tierney, Nia Roberts, Veronika Williams, Kamal R. Mahtani

**Affiliations:** 1 Nuffield Department of Primary Care Health Sciences, University of Oxford, Oxford, UK; 2 Bodleian Health Care Libraries, University of Oxford, Oxford, UK; 3 School of Nursing, Nipissing University, North Bay, Canada

**Keywords:** Primary health care, Paramedic, Realist review, Extended roles, Additional roles, Allied health personnel, Ambulatory care, Urgent care

## Abstract

**Background:**

Since 2002, paramedics have been working in primary care within the United Kingdom (UK), a transition also mirrored within Australia, Canada and the USA. Recent recommendations to improve UK NHS workforce capacities have led to a major push to increase the numbers of paramedics recruited into primary care. However, gaps exist in the evidence base regarding how and why these changes would work, for whom, in what context and to what extent. To understand the ways in which paramedics impact (or not) the primary care workforce, we conducted a realist review.

**Methods:**

A realist approach aims to provide causal explanations through the generation and articulation of contexts, mechanisms and outcomes. Our search of electronic databases was supplemented with Google and citation checking to locate grey literature including news items and workforce reports. Included documents were from the UK, Australia, Canada and the Americas—countries within which the paramedic role within primary care is well established.

**Results:**

Our searches resulted in 205 included documents, from which data were extracted to produce context-mechanism-outcome configurations (CMOCs) within a final programme theory. Our results outline that paramedics are more likely to be effective in contributing to primary care workforces when they are supported to expand their existing role through formal education and clinical supervision. We also found that unless paramedics were fully integrated into primary care services, they did not experience the socialisation needed to build trusting relationships with patients or physicians. Indeed, for patients to accept paramedics in primary care, their role and its implications for their care should be outlined by a trusted source.

**Conclusions:**

Our realist review highlights the complexity surrounding the introduction of paramedics into primary care roles. As well as offering an insight into understanding the paramedic professional identity, we also discuss the range of expectations this professional group will face in the transition to primary care. These expectations come from patients, general practitioners (family physicians) and paramedics themselves. This review is the first to offer insight into understanding the impact paramedics may have on the international primary care workforce and shaping how they might be optimally deployed.

**Supplementary Information:**

The online version contains supplementary material available at 10.1186/s12916-021-02019-z.

## Background

Paramedics within the United Kingdom (UK) are traditionally associated with the provision of emergency care within an emergency medical service (EMS), responding to life-threatening emergencies through the 999 call system. However, over the last decade, changes to healthcare access for patients have created a sociocultural dependence on EMS [1]; now, only 8% of 999 calls are for life-threatening illnesses or injuries [2], indicating that a large proportion of patients access EMS with lower acuity presentations. As the care provided by EMS has changed, the role of the paramedics has subsequently evolved. As well as advanced life support, paramedics now need to be skilled in managing long-term conditions, acute presentations of mental ill-health, social-care assessments and a range of urgent care presentations [3–5]. For the UK, this expanded role for paramedics to focus on urgent care has coincided with a move to degree-level pre-registration programmes [6], and a career framework for paramedics to progress in specialist practice in urgent or critical care, before moving onto more generalist advanced roles through postgraduate study [7]. Whilst the UK has been at the forefront of the professionalisation of paramedics globally, similar changes to EMS in other high-income countries (such as Australia, Canada and the United States of America (USA)) have prompted a similar development of the paramedic role to include provision for urgent, as well as emergency, calls. This has also coincided with the professionalisation of the paramedic profession in these countries, including graduate entry for paramedics in Australia and Canada [8] and regulation for Australasian paramedics [9].

As the paramedic profession has steadily evolved, primary care workforces have simultaneously undergone significant changes. With an increased demand in services, and more patients requiring complex case management within the community, primary care services are facing unprecedented challenges [10]. These challenges are leading to recruitment and retention issues for doctors within primary care [11], requiring workforce changes and opportunities for other clinicians to work in this setting to support general practitioner (family physician) roles [12, 13]. Attracted to ‘normal hours’ and an opportunity to further develop their practice [14], the professional evolution of paramedics within EMS has equipped them to be well suited to work in primary care. Therefore, it is unsurprising that they are a professional group welcomed to this clinical setting [15, 16].

The literature from Australia, Canada and the USA also features examples where paramedics are employed by local EMS to provide primary care services. Examples include community outreach/first aid posts [17, 18], preventative or rehabilitation services for vulnerable patient groups [19] or to expand the reach of health services to ‘medically underserved’ [20]. These activities were traditionally done by existing healthcare professionals in primary care, such as general practitioners (GPs) or nurses. However, recruitment difficulties mean that paramedics have been employed to provide such services.

Whilst paramedics may be primed to work well in primary care, as they transition into these roles, their knowledge and skillset will undoubtedly change [3, 4, 13]. Our recent scoping review of evidence published since 2005 [21] outlined that paramedics can safely apply their extended skills to assess and treat patients in primary care, but there were conflicts in relation to job titles, roles and responsibilities. Despite this, the role was received positively by patients. This scoping review outlined the lack of standardisation and complexity of the role of paramedics in primary care and that paramedics working in primary care are most helpfully conceptualised as a complex intervention. Understanding complex interventions requires a clear theoretical model outlining the contributing components and how these work together to produce outcomes [22], which are context-sensitive. The factors that underpin how paramedics work well (or not) in primary care are unclear and likely to depend on a range of different contexts.

This realist review builds on the aforementioned scoping review [14] to offer an in-depth understanding of how paramedics might work in practice, for whom, in what circumstance and how to optimise the contribution of paramedics to primary care. Realist reviews are a theory-driven approach to evidence synthesis. They are underpinned by a realist philosophy of science in which causation is viewed as a generative process where the outcomes are caused by context-sensitive mechanisms [23]. Outcomes in complex interventions are explained by context, mechanism, outcome configurations (CMOCs), where certain mechanisms are triggered by certain contexts, producing certain outcomes. These come together to create a programme theory about how an intervention is thought to work, and under which conditions [24]. Reviewing the evidence concerning paramedics working in primary care in this way is warranted given the prominence of paramedics working in primary care globally.

## Methods

**Fig. 1:**
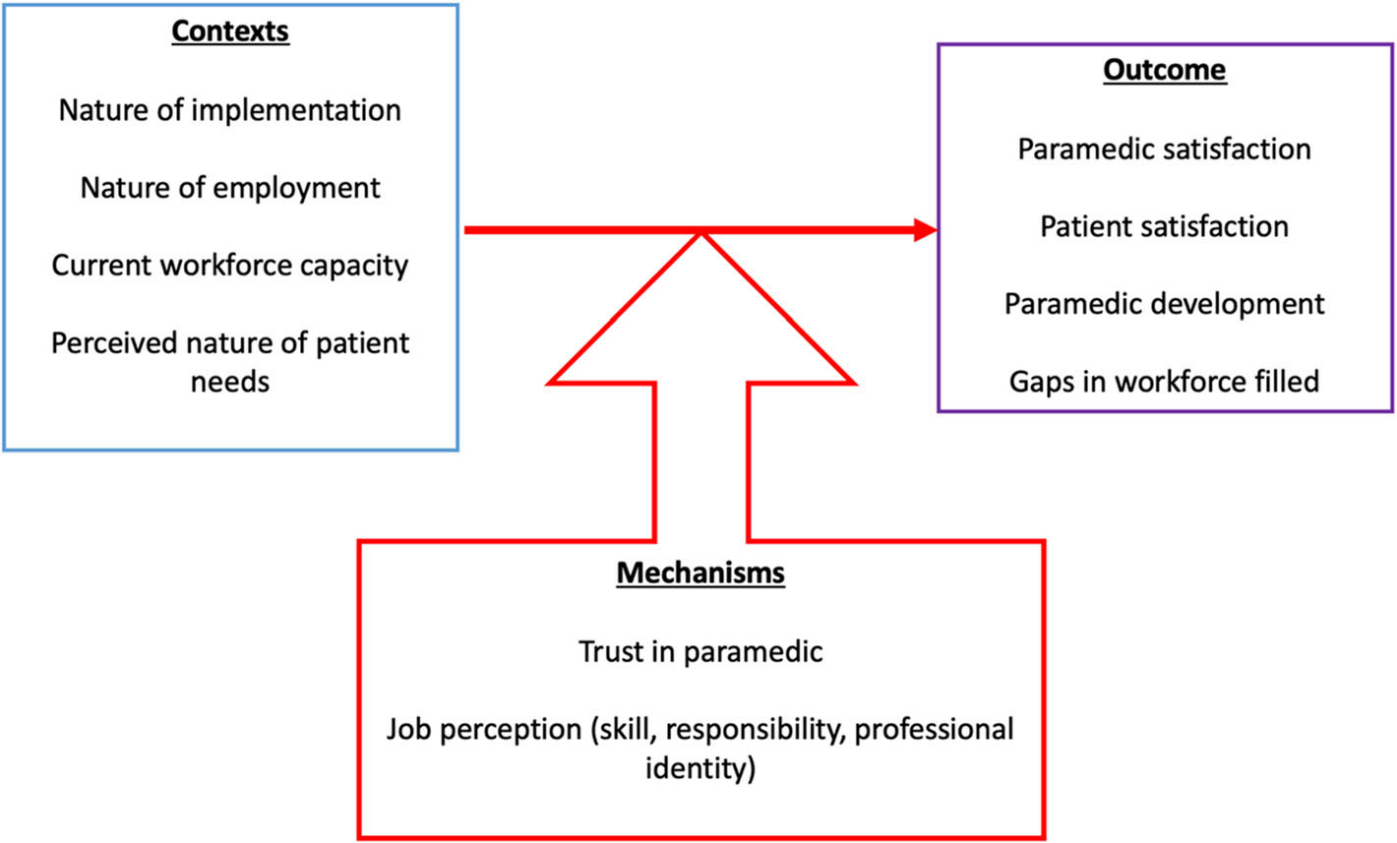
Initial programme theory

We used our exploratory scoping of the literature [21] to develop an initial programme theory (Fig. 1) to explain how paramedics work in primary care. Beginning with this theory, and through discussions with our patient participatory group (*n* = 8) and representatives from key stakeholder groups (*n* = 6), we focussed on a subset of themes that seemed most relevant in understanding this complex intervention.

Our review is reported following the RAMSES publication standards for realist synthesis [25].

### Step 1: Searching process

We built on searches designed during our systematic scoping review, which were piloted and refined with the help of an information specialist. Cochrane Database of Systematic Reviews [29/01/2021], MEDLINE (OvidSP) [2002-29/01/2021], CINAHL (EBSCOHost) [2002-29/01/2021], PsycINFO (OvidSP) [2002-29/01/2021], Embase (OvidSP) [2002-29/01/2021], NHS EED and DARE via CRDWeb (https://www.crd.york.ac.uk/CRDWeb/) (01/01/2002 to 29/01/2021), ERIC (ProQuest), Joanna Briggs Institute (https://jbi.global/), EBP (https://jbi.global/ebp) and OpenGrey (http://www.opengrey.eu/) databases were searched using free-text keywords and subject headings for the two key concepts: paramedic and general practice/primary care. An additional search of Google was undertaken with adapted keywords, where the first ten pages of results were reviewed (see Additional file 1). The citations of the screened articles were also reviewed for any new publications not found within the searches.

Our previous scoping review limited the findings to paramedics working in the UK [21] but was expanded for this realist review to capture additional relevant papers. These included papers were authored in countries in which the paramedic profession is most similar to that of the UK (through either education or regulation) and where paramedics working in community roles are occurring [26–29]. Table 1 outlines the inclusion and exclusion criteria used during the searching process. Whilst UK paramedics were first known to be taking up positions in primary care in 2002 [30], there were no empirical papers on paramedics in the UK before 2004. Articles were first searched from January 2004 to March 2019. The search strategy was repeated in April 2020 and January 2021 to determine the presence of any new articles following significant events for the paramedic profession, such as independent prescribing legislation change in the UK in 2019 [19] and updates to the English GP Contract in 2020 [31].

**Table 1:** Inclusion and exclusion criteria

Inclusion	Exclusion
• Written in English • Situated in primary care (minor injury unites, out-of-hours services, urgent care centres, walk-in centres, first aid units, general practice, family practice clinics) • Paramedic (Australia, Canada, UK, USA)	• Countries where paramedics were not state registered or educated to the same standard as the UK

The formal search resulted in 4446 articles, after duplicates were removed via the reference management software (Mendeley version 1.19.8). Retrieved literature for screening included journal publications, policy, stakeholder analysis, workforce reports, conference proceedings, case studies, job advertisements and opinion pieces.

### Step 2: Selection and appraisal of documents

**Fig. 2:**
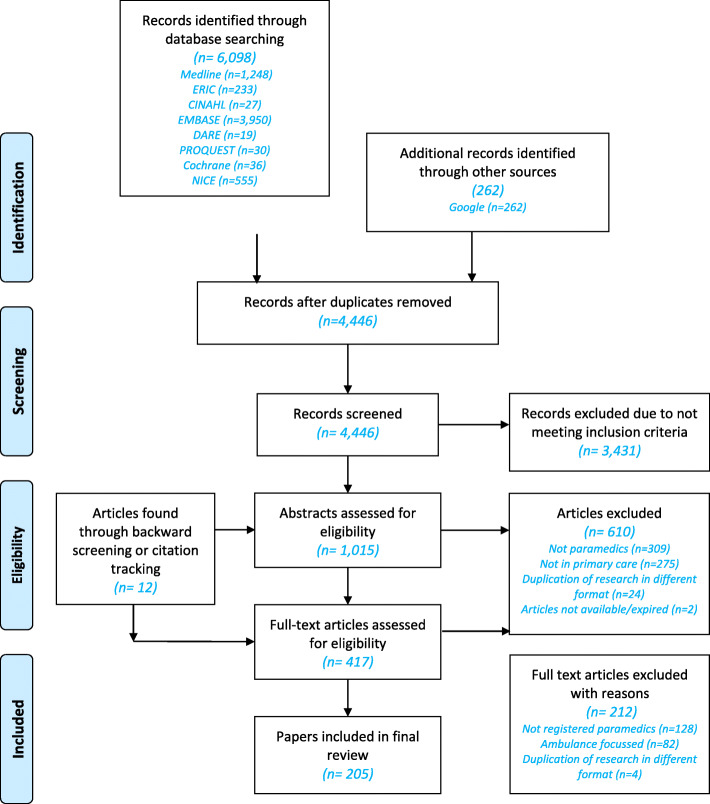
Document selection and appraisal flowchart

Screening of articles was undertaken in two phases by GE—firstly by title and abstract, and then by full text. At both phases, the inclusion and exclusion criteria outlined in the protocol [32] were used (see also Fig. 2). Full-text articles assessed for eligibility were read and checked to see if they contained relevant data that were of sufficient rigour [33]. Data were considered relevant if contributing to the development or testing of the emerging CMOCs within the programme theory. Of the 205 articles included in the review, 20% were checked for consistency by another researcher, with two articles prompting discussion for inclusion regarding the rigour of the methodology used. These were included following further discussion with GW.

### Step 3: Data extraction and organisation

Document characteristics were extracted into an Excel spreadsheet and included full-text documents uploaded into NVivo for data management and coding by GE. Coding was initially inductive, classifying content into abstract categories, such as education, scope of paramedic role and perceptions of paramedics. Ten per cent of these initial codes were viewed independently by another researcher.

### Step 4: Synthesising evidence and drawing conclusions

Following the sorting of data into abstract categories, and (where possible) potential contexts, mechanisms and outcomes within each category, a realist logic of analysis was applied to develop CMOCs that explain how an outcome was caused by the interaction between the context and mechanism. We used the process set out by Papoutsi et al. for operationalising a realist logic of analysis [34]. This was repeated for all the data found within each abstract category [35]. It enabled sets of potential CMOCs to be built by GE that started to explain the factors affecting how paramedics work in primary care [36].

Discussion of the potential CMOCs built by GE took place between GW, ST, VW and KRM, throughout the review. This continued until CMOCs were able to account for the wide range of outcome patterns found within data from included documents.

#### Discussion with stakeholders and members of the public

CMOCs were presented to our patient participation group and representatives from key stakeholder groups including paramedics and GPs working in primary care, the College of Paramedics, Health Education England, the Nuffield Trust and the Royal College of General Practitioners. Discussion with individuals was used to confirm, refute or refine the CMOCs and to develop an understanding of how and where these fitted into the programme theory.

#### Engagement with substantive theory

Consideration was given to any substantive theories that were referred to within the articles. As the CMOCs and programme theory developed, we considered a range of existing theories that could further our understanding of emerging findings. Throughout data organisation and synthesis, we sought links between the emerging CMOCs and existing substantive theories, in order to deepen our understanding regarding how paramedics work in primary care and increase the usefulness of the developing programme theory overall.

## Results

**Fig. 3:**
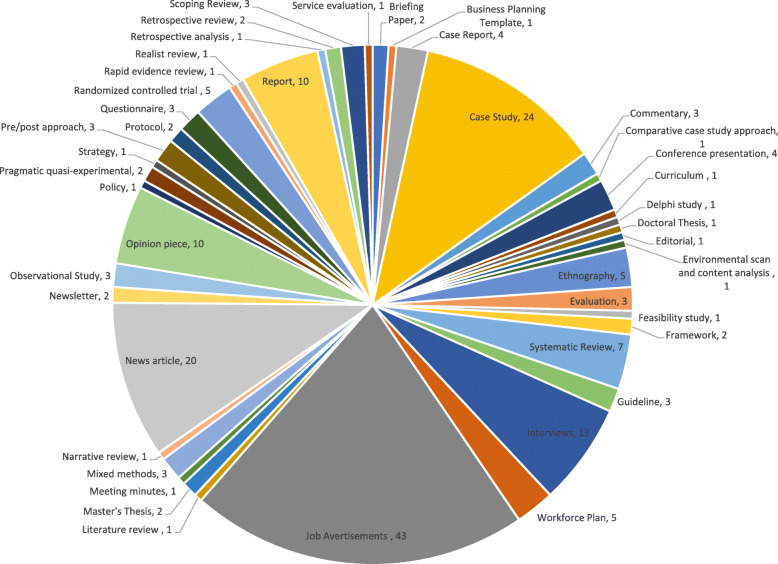
Types of document pie chart

In total, 205 documents were coded to refine our initial programme theory and develop CMOCs. Documents published between 2004 and 2021, covering paramedics working in primary care roles across Australia, Canada, England, Finland, Scotland, Wales and the USA, are outlined in Additional file 2. As illustrated in Fig. 3, most documents used to develop the CMOCs were from case studies, job advertisements, news articles, workforce evaluations or reports.

In this section, we provide a narrative overview of three key abstract categories that emerged from the 28 CMOCs developed from these documents to produce our final programme theory about how paramedics work in primary care roles (Additional file 3).

### Abstract category 1: Expectations of paramedics working in primary care

Whilst the paramedic role may be well established within some systems, such as the UK NHS, understanding the expectations of how paramedics may contribute and work within primary care was considered to be important within the literature we found. These expectations were considered in the light of patient perspectives and professional perspectives from GPs and paramedics, as well as framing the contribution of paramedics within local workforces.

#### Patient perspectives

Patients may view the role of the paramedic in primary care favourably after being informed of it by a trusted source, such as their own GP or national communications from the health system. Uncertainty exists when the role of the paramedic is not made clear to patients or their expectation is not met if they attend an appointment with a paramedic when they believed they were seeing their usual GP.

In some reviewed literature, patients expressed their initial confusion at being seen by a paramedic in the primary care setting—concerned that their problem was considered to be an emergency by the provider. However, when patients became more familiar with the role, trusting the credibility of the paramedic by their employment within primary care, high satisfaction rates were reported.

There was evidence to suggest that paramedics had a longer consultation time than their GP colleagues, and patients responded positively to this as they valued the opportunity to discuss their problem with less time constraints.

#### GP perspectives

Across each country included in this review, there was evidence of ‘early adopters’ within primary care—GPs who could see the potential for paramedics in their traditional role to contribute to the primary care workforce. For these early adopters, paramedics were viewed positively by GPs and were associated with reducing their workload and saving time in patient access to appointments. In a similar way, GPs who worked alongside their local paramedics (such as when referrals were made for patients to be reviewed by GPs following paramedic attendance through EMS) gained insight into an individual paramedic’s capabilities and subsequently offered them employment as they recognised that their skills were useful to the team.

Whilst there was much positivity when considering the paramedic in primary care from the perspective of the GP, in some reviewed literature, GPs saw paramedics as offering an ‘eyes and ears’ approach only. Using them for assessment-only roles, paramedics were not regarded as autonomous clinicians who would be able to diagnose and manage patients on their own, and thus required clinical oversight from a GP. Deployment of paramedics in such a way was unlikely to free up GP time and often led to unintended consequences such as patient frustration in the unnecessary duplication of consultations.

#### Paramedic perspectives

The literature outlined that paramedics perceive themselves as generalist clinicians who, by virtue of their work within the EMS, need to respond to all types of patients, across all ages, with any presenting complaint. Due to their generalist nature, paramedics would seek opportunities to work in primary care, believing their capabilities would fit well within this workforce.

In a similar way, paramedics consider employment in primary care as an opportunity to develop their existing skillset within a structured, supported environment, which is in contrast to emergency service culture. The opportunity to build relationships with patients, rather than engage in one-off episodes of care was considered professionally fulfilling. Overall, working in primary care was associated with a better work/life balance in terms of no nightshifts and a community-focussed working environment.

#### Contribution to primary care teams

The idea that paramedics were pluripotential was considered a useful addition for primary care teams, where they had the capabilities to deal with a breadth of issues, as well as being developed to a narrower focus as the setting demanded. However, where the skills and competencies of the paramedic were not suitable for primary care (such as when urgent assessment clinics were already being run by another discipline, such as nurses), paramedics were not considered to be a useful addition to the team.

There was evidence to suggest that paramedics working in primary care roles make a difference in environments where access to healthcare otherwise would not be available or delayed, such as in rural communities. Examples of this were typically found where paramedics were employed by EMS to manage and run first contact centres, which could see the range of conditions associated with primary care, as well as more urgent presentations that would be more associated with work undertaken by EMS. Such workforce ‘rotational’ models were highly valued by commissioners, employers, paramedics working in them and the patients who benefited from improved healthcare access.

Within the English literature published after the introduction of the *Additional Roles Reimbursement Scheme* in 2020 (where primary care employers are reimbursed for the employment of paramedics), paramedics were more widely considered to be a credible addition to the local primary care workforce, as they were regarded as having been endorsed by trusted organisations (such as NHS England).

### Abstract category 2: Transition from EMS into primary care roles

Some evidence suggested that paramedics can transition into primary care (particularly to advanced practice roles) when supported by primary care (e.g. getting access to formal education and clinical supervision within the workplace).

#### Education

The need to build upon existing skills and competencies for paramedics to be more effective in primary care was considered across many of the case study and evaluation literature. The clinical gaps that need to be filled for a successful transition to primary care centred around biochemistry (for the understanding and interpretation of blood tests), pharmacotherapy (to support independent prescribing for long-term conditions or complex patient groups) and some technical skills such as wound care, urinalysis and imaging.

Throughout the literature, higher levels of paramedic education were associated with a higher level of pay and an increased scope of practice and clinical responsibility. Such attainment was used as a marker to differentiate between advanced paramedic roles at master’s level education and first contact/community/extended paramedic roles.

#### Supervision

The success of the transition to primary care from EMS was linked to the provision of supervision to support paramedic clinical development. Clinical supervision enabled paramedics to feel supported as they adjusted their skillset to a new clinical setting and gave them confidence and satisfaction in their new role. Supervision also enabled GPs to build up trusting relationships with the paramedics, who could then be accepted into the primary care team.

Where clinical supervision was not provided, or where there were difficulties in the supervisory relationship, paramedics reported feelings of isolation and lower satisfaction with the work in their role, opting to return to EMS employment.

#### Experience

Throughout the literature across all countries, an arbitrary 5 years of post-registration experience within EMS was considered a requirement for paramedics entering primary care roles. Role consolidation was important for policymakers, employers and paramedics, all of whom made links between the length of exposure to patients as an autonomous clinician within EMS and successful transition into primary care.

### Abstract category 3: Role and responsibilities

When considering the factors that affect the integration of paramedics into the primary care team, the literature suggests that when the role or responsibilities are unclear, there is dysfunction in the employment of paramedics in primary care.

#### Working in a team

The need for integration into the primary care team was crucial to avoid both role duplication and role substitution. Both were less likely to occur when the professional role boundaries of the paramedic in primary care did not overlap with existing healthcare professionals, and where paramedics were aware of their own professional competencies. However, where role boundaries became blurred, or where the paramedic was viewed as *Johannes factotum* (or jack-of-all-trades), the literature suggests that resistance to paramedic roles was due to a lack of trust from other healthcare professionals, or other healthcare professionals feeling threatened or disempowered due to the implementation of these new roles alongside the existing ones.

#### Interpersonal skills

The ability of paramedics to build rapport and trusting relationships in a short amount of time (as required during emergencies) was considered an important component for replication in primary care. Patients were more satisfied when attended by paramedics with strong interpersonal skills and enthusiasm, citing their ability to connect to these healthcare professionals as a key marker of the success of their work in primary care. GPs also saw these interpersonal skills as crucial to match the values held by the GPs, leading to the integration of specific interpersonal skills into the essential criteria of job descriptions advertising for the role.

### Contribution of existing theory

**Fig. 4:**
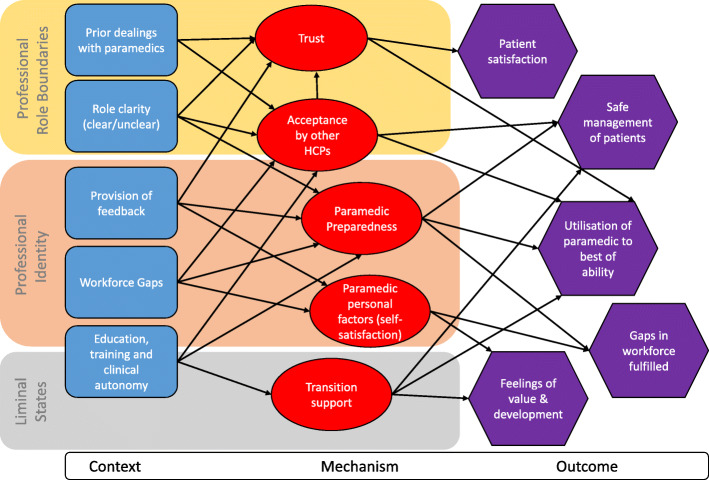
Final programme theory and substantive theory

Our engagement and incorporation of substantive theory to develop our CMOCs followed an abductive process to elaborate on the proposed mechanisms and continue the process of refinement until the programme theory became more nuanced. We drew on the following substantive theories: *professional role boundaries*, *professional identity* and *liminal state*. Integration of these with our programme theory is illustrated in Fig. 4.

#### Professional role boundaries

Cultural sociologists suggest that group boundaries are shaped by institutionalised definitions of cultural memberships [37], thus enabling an understanding of how professions come to be distinguished from one another. The notion of a ‘profession’ originally emerged as a demarcation problem between ‘superior’ and ordinary occupations, where the former could be defined by their particular knowledge base, education, credentialling and autonomy [38]. Such a trait approach emphasises the monopolistic nature of social boundaries between different professional groups, where each profession is a clearly bordered unit developed from a functional specialisation, ‘within which a formal body of knowledge and skill can develop, be nourished, practised, refined and expanded’ [39]. Whilst such closed models (for example, between physicians and paramedics) exist, professions are also considered to exist in an interdependent open system whereby there is competition for jurisdictional monopolies and the legitimacy of the claimed expertise [40]. Applied within one setting (such as primary care), this leads to a constantly changing system of professions, with disputes on the social boundaries between them. This was seen in the literature reviewed, where the concept of role substitution, rather than workforce addition, was a commonplace concern for GPs and other clinical staff within primary care. This was expressed around the clinical role and contribution paramedics should or could be undertaking and compared against other ‘traditional’ primary care healthcare posts, such as nurses.

#### Professional identity

In considering how paramedics view themselves and are viewed by patients and other healthcare professions, we draw on theories of professional identity from Freidson [39]. Whilst such a trait approach may now be considered an inappropriate way to define professions [38], it remains important due to the considerable contribution it has made to academic debate. It also highlights how professions have been viewed historically, which is important when trying to understand the attributes to which occupations may have been expected to aspire in order to become professions.

Knowledge, uncertainty and discretion [39] are essential elements in the work for healthcare professionals, and trust in the cognitive authority of the paramedic is needed to enable them to be accepted into the primary care environment. This discretion is given to the paramedic based on trust that the paramedic will use their knowledge and skills in the best interest of the patient and that they are not only morally involved, but also involved from a point of regulation. Within the literature we reviewed, paramedics were accepted into primary care workforces (or not) based on perceptions of their professional identity by GPs. In a similar way, paramedics chose to enter employment in primary care when they were comfortable with their professional identity and the contribution they could make within the workforce team.

#### Liminal states

In the literature reviewed, difficulties were encountered for paramedics transitioning into primary care roles when there was a lack of understanding of the range, purpose or responsibility within the new role. Moving into primary care can be viewed as a threshold concept, where there are key changes to the way in which the discipline is practised and without understanding of which the clinician cannot progress or transition [41]. Until these threshold concepts have been grasped, then paramedics span a precarious existence where they are no longer associated with their traditional role in EMS but have not yet made a full transition into primary care. This is best described as a state of liminality, in which there is only a partial understanding of how they ‘work’ in their new role [42]. This existed in instances within the literature that described a lack of empowerment for the paramedic to be autonomous in their practice, where they worked within a model of decisive medical oversight, rather than support.

### Final programme theory

Our final programme theory (Additional file 3) shows that paramedics are more likely to be effective in contributing to primary care workforces when supported to develop their knowledge through formal education (such as a postgraduate degree) combined with clinical supervision within the primary care setting. This also builds trust between the paramedic and GP and helps the paramedic to find their role within the workforce, without threatening the contributions of other professions. Paramedics who are trusted to practise at their full potential are more satisfied working in primary care, and this may contribute to the enthusiasm perceived by patients in their role. Paramedics with strong interpersonal skills are highly rated by patients, and the development of a trusting relationship between patient and paramedic is paramount in meeting patient expectations, but also acceptance of the role. In order for patients to accept paramedics in primary care, the role and its implications for their care should be outlined by a trusted source (such as the primary care clinic or surgery); when this is done, it engenders support for these new roles.

Understanding about the deployment of paramedics into primary care roles was also gained from the literature. Paramedics were able to integrate well within primary care and EMS when they worked in a rotational role. This was attractive from a personal professional identification point of view, as well as by EMS who otherwise would risk losing their most experienced and highly educated staff. Such a peripatetic nature may not enable paramedics working in such a way to be embedded or socialised enough in primary care or socialised enough to build trusting relationships with patients or GPs. However, paramedics employed by EMS providing primary care services in remote settings were able to address healthcare access gaps and were embedded within local communities accessing these services.

## Discussion

Paramedics have been increasingly established in primary care over the last decade in a number of countries. Our review of policy documents, workforce evaluations, case studies and primary research suggests that benefits associated with paramedics working in primary care settings include a reduced GP workload, better access to health assessment and care for patients and career development for this group of professionals outside of their traditional EMS employer. This is the first published systematic synthesis of the literature, using a realist lens, to explore how this role can be implemented optimally.

This review has drawn on 205 documents to present a programme theory outlining how paramedics may currently be working in primary care and the extent of their contribution in these roles. Our programme theory proposes that paramedics entering primary care need to navigate complex professional role boundaries in order to establish their professional identity and contribute to the primary care workforce. Desired outcomes, such as providing an addition to the primary care team (and perhaps reducing GP workload), may then transpire. In order for paramedics to work successfully as part of the primary care team, they need to transition effectively in these roles, supported through formal education to fill the knowledge gaps and clinical supervision to build trusting relationships with GPs. For paramedics working in rotational roles between primary care and the EMS, their peripatetic nature means that they may often practise on the periphery of both settings and, consequently, have a weaker connection to the organisational or professional norms and values, limiting their development and contribution.

Recent guidance published by Health Education England has produced a ‘roadmap’ for paramedics to follow as they transition into primary care roles [43]. This has helpfully outlined specific qualifications, skills and aptitudes for two tiers of paramedics working in primary care: first contact practitioners and advanced practitioners. The findings of this review offer additional dimensions for consideration. For example, whilst we have found that interpersonal skills of the paramedic are important, consideration of the patient perspective is also needed. Our review highlighted the importance of patient understanding of this new role working in primary care in building acceptance, trust and confidence in being seen by clinicians other than their usual GP.

### Strengths and limitations

This realist review was conducted systematically and transparently, in accordance with the RAMSES quality standards [44]. The CMOCs and programme theory were developed through regular team discussions, as well as contributions in the form of feedback and advice from patients and members of the public and representatives of key stakeholder groups. The programme theory and its embedded CMOCs have an analogy with three substantive theories. Our authorship team represents a variety of clinical and academic backgrounds, ensuring divergence in our analysis.

Limitations include our analysis on publicly accessible literature, located through recognised research databases and Google. Whilst we found workforce reports and case studies through our searches, these do not account for similar documents that undoubtedly exist within organisations, but which have not been made publicly available. Many of the documents found in this review were evaluation, case study or opinion. Where primary research was included, these were not without methodological limitations that affected either reliability or transferability of the reported results. Such data may not be considered reliable in a traditional hierarchy of evidence, but by drawing our interpretations from data contained within multiple documents, we were able to develop explanatory theories that had plausibility [45]. Whilst this has enabled us to make the knowledge claims set out in our programme theory, this should be interpreted with caution until additional primary data collection can confirm, refute or refine parts of this theory. Such data collection should address the gaps that our theory presents, such as the experience needed for the paramedic to contribute efficiently to primary care, or whether standardisation of this role can exist within regulatory boundaries. Lastly, some of our evidence is authored from Australia and the Americas. Whilst similar education and scope of practice exist between paramedics in these settings to their UK counterparts, there are differences in the standardisation of practice, regulation and overall role contribution to healthcare. However, our interpretation of the literature we reviewed is that there are more similarities for paramedics working in primary care within these countries, compared to work they undertake in EMS. Hence, the inclusion of data from such documents helped us to develop our CMOCs.

### Implications for practice and policy

**Fig. 5:**
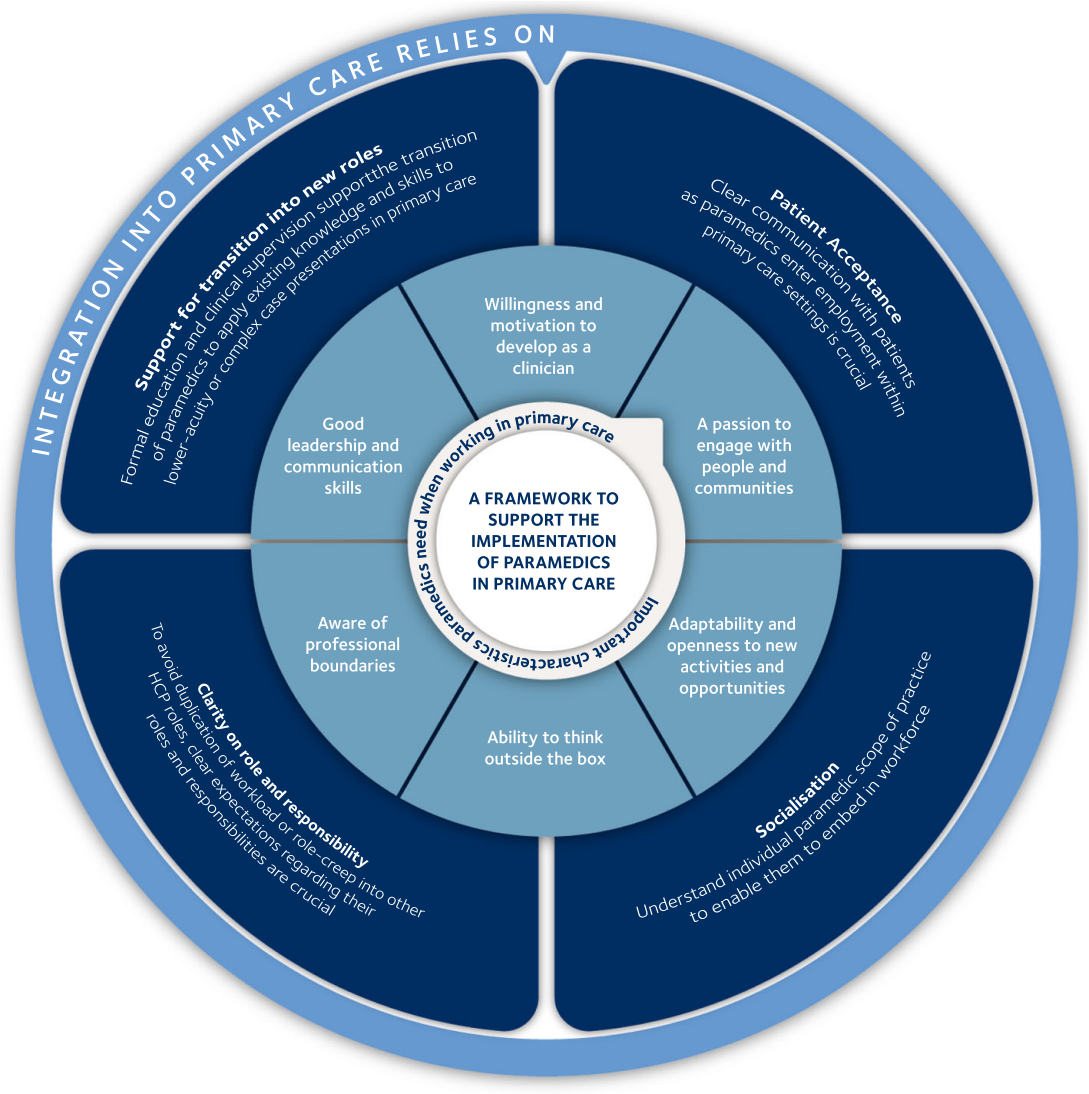
A framework to support the implementation of paramedics in primary care

Our review outlines the mechanisms that are triggered when paramedics work in primary care roles and the range of contexts that exist within these roles that trigger these mechanisms. In particular, we identify a range of outcomes, some of which differ from predicted desired outcomes that the implementation of paramedics into the primary care workforce seeks to have from a policy perspective [46]. This has potentially important implications for England and possibly wider afield, where the recruitment of paramedics into primary care roles is a key component of the proposed workforce strategy [12, 15]. Based on this realist review, the employment and integration of paramedics into primary care should consider the following, which is also summarised in Fig. 5.

#### Patient acceptance of the paramedic role in primary care

Patients need to develop confidence and trust in seeing paramedics in primary care. There is a risk of confusion and frustration for patients who expect to see their GP but are seen by a paramedic instead, especially if patients do not understand the paramedic role. Therefore, clear communication with patients, as paramedics enter employment within primary care settings, is crucial. This could be done at both a local or national level and needs to come from a respected source for patients to accept paramedics.

#### Socialisation of the paramedic role into the primary care team

To contribute effectively to the primary care setting, paramedics need to be embedded within the workforce. Understanding the individual paramedics’ scope of practice is important as this impacts how the additional role can most effectively contribute. Being embedded within the workforce also fosters trust between paramedics and other healthcare professionals, and paramedics are more likely to be satisfied with their role. This is imperative for paramedics who are employed in rotational models between two clinical settings (such as EMS and primary care), to ensure they can become effective team members within both settings.

#### Clarity regarding role and responsibilities for paramedics

For paramedics to be accepted by other healthcare professionals in primary care settings, clear expectations regarding their roles and responsibilities are crucial. When paramedics are not used to the best of their ability, patients may experience duplicate consultations, and paramedics are frustrated by a lack of autonomy. Understanding the role and responsibilities of the paramedic also needs to be in consideration of other healthcare professionals employed in the setting, to avoid repetition of workload or role-creep into other healthcare professionals’ roles, such as nursing.

#### Support for transition into new roles

Whilst paramedics are generalist clinicians, this is in the context of emergency situations. Paramedics will need support to apply their existing knowledge and skills to lower-acuity or complex case presentations. Support for transition into primary care roles could be in the form of formal education (such as a master’s degree) and/or the provision of clinical supervision to support their practice development. Equally, paramedics need to have an awareness of personal and professional limitations in order to seek support when required to benefit patient care.

#### Self-awareness

Paramedics may be considered as both health advocates and emergency experts. The ability to build rapport and trust with patients is a key component of emergency care, which transfers well into primary care. The criteria for paramedics being able to successfully embed within their new roles, contribute to the workforce capacity and reassure patients include the following:

- A passion to engage with people and communities
- Adaptability
- Ability to think outside the box
- Being aware of professional boundaries and drawing on the expertise of colleagues when required
- Good leadership and communication skills
- Willingness and motivation to develop as a clinician

### Implications for research

Our final programme theory has highlighted the areas requiring further investigation in order to determine the contribution paramedics can make to primary care. These include the following:

- How a paramedic can best transition into primary care roles from EMS and the education they require to fill in knowledge gaps and to work efficiently in this new practice setting
- The duties undertaken by paramedics working in primary care, without causing duplication, substitution or boundary disputes with existing primary care roles
- Whether paramedics maintain their existing professional identity as they move into primary care and whether this is required for them to work in primary care
- Exploration of which specific patient groups paramedics may be best targeted when working in primary care

The evaluation of cost-effectiveness existed in some of our included literature that we have not focussed on in this review. This area necessitates separate analysis, especially in consideration of how difficult it is to assess cost-effectiveness in a complex intervention with a range of outcomes [47].

## Conclusion

Despite paramedics being well established in primary care roles across the UK, Australia, Canada and the USA, there has existed a paucity of understanding regarding ‘how’ these roles work to contribute to primary care workforces and the patients that they see. Our realist review highlights the complexity surrounding the introduction of paramedics into primary care roles. As a complex intervention, the work that paramedics undertake in primary care should have a strong theoretical underpinning that can account for how they work, why they work and for whom they work best in order to guide practical deployment. We have developed a programme theory for this purpose. Our programme theory highlights that a key element for paramedics to be able to work efficiently in these roles is formal education and clinical supervision to support and develop their decision-making and autonomy. Such support enables the transition of the paramedic from EMS to the primary care setting and supports them to navigate their professional role boundaries and develop their professional identity. As well as offering an insight into understanding the paramedic professional identity, we highlight the range of expectations this professional group will face as they transition into primary care, coming from patients, GPs and paramedics themselves. This is the first published review to offer insight into understanding the impact paramedics may have on the primary care workforce, and we offer indicative gaps that need addressing if the implementation of these healthcare professionals is to be effective and productively contribute to primary care.

## Supplementary Information

**Additional file 1.** Search Strategy.

**Additional file 2.** Tables of Studies.

**Additional file 3.** Final Programme theory.

## Data Availability

Datasets used in the review are available from the corresponding author upon request.

## Abbreviations

CMOCs: Context-mechanism-outcome configurations
EMS: Emergency medical services
GP: General practitioner
NHS: National Health Service
UK: United Kingdom
USA: United States of America
