# Supplementary material for: Understanding the role of the paramedic in primary care: a realist review
Source: BMC Med. 2021 Jun 25;19:145. doi: 10.1186/s12916-021-02019-z (PMC8229679; doi:10.1186/s12916-021-02019-z)
Supplement: Supplementary file 1 — Additional file 1. Search Strategy. [file 12916_2021_2019_MOESM1_ESM.docx]

**Additional File One**

**Search strategy: Medline (Ovidsp)**

1 Allied Health Personnel/ and emergenc*.mp.

2 Emergency Medical Technicians/

3 (paramedic* or ((emergency or ambulance) adj3 (technician? or practitioner? or staff* or personnel or workforce))).tw.

4 1 or 2 or 3

5 exp General Practice/

6 general practitioners/ or physicians, family/ or physicians, primary care/

7 Primary Health Care/

8 Community Medicine/ or Community Health Services/ or Rural Health Services/

9 After-Hours Care/

10 Ambulatory Care Facilities/

11 Office Visits/

12 ((family or general) adj3 (practi* or doctor? or physician?)).tw.

13 (primary adj (care or healthcare or "health care")).tw.

14 (community adj2 (care or medicine or service?)).tw.

15 ("out of hours" or ooh or walkin or walk-in).tw.

16 ((health* or medical or ambulatory) adj2 (centre? or center? or clinic?)).tw.

17 *Triage/

18 triage.ti.

19 (Remote Consultation/ or Triage/) and Telephone/

20 exp Call Centers/

21 (helpline? or help line? or hotline? or hot line? or call centre? or call center?).tw.

22 (telephone? adj3 (service? or centre? or center? or triage)).tw.

23 ((enhanc* or expand*) adj3 role?).tw.

24 5 or 6 or 7 or 8 or 9 or 10 or 11 or 12 or 13 or 14 or 15 or 16 or 17 or 18 or 19 or 20 or 21 or 22 or 23

25 4 and 24

26 ((community or primary care or primary health care or primary healthcare) adj3 paramedic*).tw.

27 25 or 26

28 limit 27 to yr="2004 -Current"

**Search strategy: Google**

Paramedics (“primary care” OR “general practice”) site:nhs.uk
