# Supplementary material for: Understanding the role of the paramedic in primary care: a realist review
Source: BMC Med. 2021 Jun 25;19:145. doi: 10.1186/s12916-021-02019-z (PMC8229679; doi:10.1186/s12916-021-02019-z)
Supplement: Supplementary file 2 — Additional file 2. Tables of Studies. [file 12916_2021_2019_MOESM2_ESM.docx]

| **Table 2a. Table of Studies** | | | | | | | |
| --- | --- | --- | --- | --- | --- | --- | --- |
| **Author** | **Year** | **Country** | **Title** | **Study Design** | **Population Group** | **Setting** | **Objectives** |
| Scott and Carney | 2004 | UK | Emergency Care Practitioners | Opinion piece | Emergency Care Practitioners working | Primary, secondary and out-of-hospital care within the UK | Discusses different considerations prior to implementation of ECP roles. |
| Hauswald et al | 2005 | USA | ﻿Expanded emergency medical services: The failure of an experimental community health program | Case Report | Extended-Emergency Medical Technicians | Rural health provision by Emergency Medical Service | Description of the implementation of E-EMTs in one rural town which identified poor clinical care and professional conduct amongst E-EMTs. |
| Misner | 2005 | Canada | Community Paramedicine: Part of an Integrated Healthcare System | Case Report | Community Paramedics work with a Nurse Practitioner in a rural area | Emergency Medical Service initiative: Community Paramedic Programme | To design an innovative delivery of primary healthcare to two isolated Nova Scotia island communities. |
| Misner | 2005 | USA | Community Paramedicine: Part of an Integrated Healthcare System | News article | Community Paramedic | Rural community paramedic roles provided by Emergency Medical Service | Description of service design and delivery of primary healthcare using community paramedics in two isolated island communities. |
| Mason et al | 2006 | UK | The evolution of the emergency care practitioner role in England: experiences and impact | Mixed methods: Questionnaire; semi-structured interviews; economic analysis | Emergency Care Practitioners (77.4% had paramedic as their root profession) | Range of clinical settings, including minor injury unit; walk-in-centre; primary care; emergency department & ambulance services | To describe the development of ECP schemes across17 sites and identify criteria contributing to a successful operational framework. Routinely collected data was analysed to provide a preliminary estimate of costs. |
| Woollard | 2006 | UK | The Role of the Paramedic Practitioner in the UK | Commentary Paper | Emergency Care Practitioners, Community Paramedic, Paramedic Practitioner, advanced paramedic practitioners | UK-informed, with population in ambulance service employment | To describe the roles and opportunities open to paramedics. |
| Halter et al | 2007 | UK | A patient survey of out-of-hours care provided by Emergency Care Practitioners | Telephone administered questionnaire | Emergency Care Practitioners (ECPs) | Out-of-hours care | The aim of the study was to evaluate the care provided to patients receiving out-of-hours home visits from ECPs in London from the patients' perspective and to assess their wellbeing following the visit. |
| Mason | 2007 | UK | Effectiveness of emergency care practitioners working within existing emergency service models of care | Mixed methods: Controlled comparative observational study; telephone interviews; economic analysis | Emergency Care Practitioners | UK 999 urban ambulance service; Rural GP-led out-of-hours service; Rural Nurse-led walk-in centre | Objectives were to evaluate the appropriateness, satisfaction and cost of ECPs compared with the usual service available in the same healthcare setting; to increase understanding of the effect ECPs were having on health service delivery; and to evaluate any cost savings achieved with ECPs work. |
| Stirling et al | 2007 | Australia | Engaging rural communities in health care through a paramedic expanded scope of practice | Semi-structured interviews with stakeholders; Review of organisational documents | Expanded Scope Paramedics | Emergency Medical Services | ﻿To explores how community engagement by paramedics in an expanded scope role contributes to both primary health care and to an overall improved emergency response capacity in rural communities. |
| Gray and Walker | 2008 | UK | Is referral to emergency care practitioners by general practitioners in-hours effective? | Service evaluation | Emergency Care Practitioners (ECPs_ | ECPs in ambulance services undertaking home visits | To evaluate the cost effectiveness to primary care trusts (PCT) in commissioning general practitioner (GP) referrals in-hours to emergency care practitioners (ECP). |
| Scottish Ambulance Service | 2008 | UK | Potential impact on preventing avoidable A&E attendances | Briefing Paper | Community Paramedics | Employed by Scottish Ambulance Service to work in General Practice | Outline some of the new ways of working by SAS staff, which impact upon avoidable emergency department attendances. |
| Blacker et a | 2009 | Australia | Redesigning paramedic models of care to meet rural and remote community needs | Conference paper (including survey results) | Extended Care Paramedic; Paramedic Community Support Coordinator; Isolated Practice Area Paramedic; Community Paramedic | Emergency Medical Services in Australia and New Zealand | To inform Council of Ambulance Authorities (CAA) member ambulance services in monitoring the development of expanding paramedic roles and to assist services in the development of their own models. |
| Dixon et al | 2009 | UK | Is it cost effective to introduce paramedic practitioners for older people to the ambulance service? Results of a cluster randomised control trial | Cluster randomised controlled trial | Paramedic Practitioners (PP) who responded to adults over the age of 60 who had fallen | Yorkshire Ambulance Service | To assess the cost effectiveness of the paramedic practitioner scheme compared with usual emergency care. |
| Mulholland et al | 2009 | Australia | Multidisciplinary Practice in Action: The Rural Paramedic – It’s Not Only Lights and Sirens | Semi-structured interviews, direct observation and document review | Advanced Paramedics | Rural provision of health run by Emergency Medical Services on the East Coast of Tasmania | ﻿This paper examines the Tasmanian portion of a four-state study commissioned by the Australian Council of Ambulance Authorities to examine the expanded scope of practice for Australian rural paramedics. |
| Coleman et al | 2010 | UK | War and peace? strategies by emergency care practitioners to integrate into health care teams in the UK | Interviews with Emergency Care Practitioners (part of the National Evaluation of Emergency Care Practitioners (NEECaP) Trial) | Emergency Care practitioners | Ambulance services; out-of-hours services; urgent care services; care homes; minor injury units that employed ECPs in the UK. | The aim of the National Evaluation of Emergency Care Practitioners (NEECaP) Trial was to evaluate the impact of ECPs on patient pathways and care in different emergency care settings. This paper focussed on the interviews undertaken with ECPs as a work package within the larger trial. |
| Mason et al | 2010 | UK | A pragmatic quasi-experimental multi-site community intervention trial evaluating the impact of Emergency Care Practitioners in different UK health settings on patient pathways (NEECaP Trial) | Pragmatic quasi-experimental multi-site community intervention trial | Emergency Care Practitioners | Ambulance services; out-of-hours services; Urgent care services; care home; minor injury units that employed ECPs in the UK | The aim of this study was to evaluate the impact of ECPs on patient pathways and care in different emergency care settings. |
| Barr, P | 2011 | USA | Doctor 911 Rural areas seek expanded roles for paramedics. | Opinion Piece | Rural Community Paramedics | Community Paramedics via Emergency Medical Services | To outline current debates regarding the implementation of community paramedic roles within emergency medical services within the USA. |
| O'Hara et al | 2011 | UK | Quality and safety of care provided by emergency care practitioners | Retrospective patient case note review | Emergency Care Practitioners | Range of clinical settings, including minor injury unit; walk-in-centre; primary care out-of-hours services; primary care home visiting services; emergency department & ambulance services | The objectives were to compare the quality and safety of care provided by ECPs with non-ECP care across different clinical settings. |
| O'Keefe et al | 2011 | UK | A community intervention trial to evaluate emergency care practitioners in the management of children | Pragmatic quasi-experimental multi-site trial | Emergency Care Practitioners (ECPs) | Urgent care, out-of-hours, minor injury units | To evaluate the impact of ECPs on the patient care pathway for children presenting with minor conditions in unscheduled care settings. |
| Ruston and Tavabie | 2011 | UK | An evaluation of a training placement in general practice for paramedic practitioner students: improving patient-centred care through greater interprofessional understanding and supporting the development of autonomous practitioners | Semi-structured interview and survey | Paramedic Practitioner students | General Practice | ﻿To report the extent to which the placement of paramedic practitioner students in accredited general practice (GP) training practices supported their development as autonomous, patient-centred practitioners and fostered interprofessional learning. |
| Wiley | 2011 | USA | Community Health Care Paramedic | News article | Community Paramedics | Emergency Medical Services | Description of the expanded role of Emergency Medical Services to provided community paramedic programmes in rural communities. |
| Ball | 2012 | UK | Setting the scene for the paramedic in primary care: a review of the literature | Literature review | Paramedics, Paramedic Practitioners: Community Paramedics; | Telephone triage services; minor injury unit; walk-in-centre; Intermediate care services | To explore the published evidence regarding paramedic practice in an attempt to identify the skills, training, and professional capacity which paramedics working in primary care will require. |
| Daly | 2012 | UK | The Paramedic in the Community: My Story | Case Study (including results of patient satisfaction survey) | Paramedic | General Practice | Overview of the role of a paramedic working in general practice. |
| North Central EMS Institute | 2012 | USA | Community Paramedic Curriculum 3.0 | Curriculum | Community Paramedic | All providers of Community Paramedics education | To train community paramedics as an international health care provider. |
| O'Meara et al | 2012 | Australia | Extending the paramedic role in rural Australia: a story of flexibility and innovation | Multiple case study methodology over one year, including semi-structured interviews, observation and review of documents which describe the paramedic role | Rural Community Paramedic models | Rural Expanded Scope of Practice (RESP) model run by Emergency Medical Services | To ﻿identify trends in the practice of rural paramedics and describe key characteristics, roles and expected outcomes for a Rural Expanded Scope of Practice (RESP) model. |
| Bigham et al | 2013 | Canada | Expanding Paramedic Scope of Practice in the Community: A Systematic Review of the Literature | Systematic Review | Community Paramedics; Paramedic Practitioner; Emergency care Practitioner | Inclusion of published literature in Australia, Canada, UK and USA | To undertake a systematic review of the international literature to describe existing community paramedic programs. |
| Evans et al | 2013 | UK | Which extended paramedic skills are making an impact in emergency care and can be related to the UK paramedic system? A systematic review of the literature | Systematic literature review | Prehospital emergency care providers (including paramedics) | Ambulance services, general practices and emergency departments in literature from Australia, Canada, UK and USA | To identify evidence of paramedics trained with extra skills and the impact of this on patient care and interrelating services such as General Practices or Emergency Departments. |
| Hill et al | 2013 | UK | A systematic review of the activity and impact of emergency care practitioners in the NHS | PROSPERO registered systematic review | Emergency Care Practitioners (ECPs). No distinction between Nursing and Paramedic professional route to ECP were outlined in the included papers | UK-only papers focussing on Emergency Care Practitioners in ambulance services, primary care and emergency departments | To summarise the national evidence-based literature on the impact of ECPs on healthcare delivery, effectiveness of practice and related health service resource use. |
| Kinney | 2013 | USA | Community Paramedic: Starting with Hospice | Case Report | Community Paramedics | Community Paramedics deployed to hospice patients via Emergency Medical Services | To outline how the programme reduces unnecessary emergency department visits during the evening hours. |
| Mettner | 2013 | USA | Keeping Emergencies at Bay | News Article | Community Paramedics | Community Paramedics undertaking home visits via Emergency Medical Services | Outline of the community paramedicine role introduced to Minnesota. |
| Nolan, M | 2013 | Canada | A Survey of Community Paramedic Programs in Ontario | Survey | Chiefs of Emergency Medical Service | Emergency Medical Service | To gather the data required for an overview of the level of Community Paramedic Program activity in Ontario. |
| Tohira et al | 2013 | Australia | The impact of new prehospital practitioners on ambulance transportation to the emergency department: a systematic review and meta-analysis | Systematic review: PROSPERO registered. | Emergency care practitioners (EmCPs); Paramedic practitioners; Extended care paramedics (ECPs) | Emergency Medical Services | To conduct a systematic review and metanalysis to examine the impact of new prehospital practitioners (NPPs), EmCPs, paramedic practitioners and ECPs, on ambulance transportation to the emergency department. |
| Drennan et al | 2014 | Canada | ﻿Expanding Paramedicine in the Community (EPIC): study protocol for a randomized controlled trial | Protocol: a pragmatic, randomized controlled trial comparing a community paramedic intervention to standard of care for patients with DM, HF and COPD. The primary outcome measure will be the rate of hospitalization at one year. Secondary outcomes will include measures of health system utilization, overall health status, and cost-effectiveness of the intervention over the same time period. | Community Paramedic | Emergency Medical Service initiative: Community Paramedicine at Home programme (home visiting) | To understand whether expanding the paramedic scope of practice reduce acute care hospitalisation. |
| Goldberg | 2014 | USA | Mobile Integrated Healthcare: Using existing out of hospital resources to bridge gaps in healthcare services | Master's Thesis: Topical Analysis | Paramedic | Mobile Integrated Health programmes (run by Emergency Medical Services) in Chicago, Indiana, Pittsburgh, San Francisco, Alaska and New Mexico | To examine the ways in which current Emergency Medical Services may use mobile integrated health programmes to bridge the gap between at-risk patient populations and health care services. |
| Hambleton, Richmondshire and Whitby Clinical Commissioning Group | 2014 | UK | Community Paramedic Practitioners | Business Planning Template | Paramedic Practitioner | Employed by Yorkshire Ambulance Service to work in General Practice | Business plan for Paramedic Practitioners to undertake home visits for primary care services as well as respond to category one calls in local area. |
| Health Education Kent, Surrey and Sussex | 2014 | UK | Clone of Paramedic Practitioner Training | News article | Paramedic Practitioner students | General Practice | Outline of the training for paramedic practitioner students. |
| Jensen et al | 2014 | Canada | Insights into the Implementation and Operation of a Novel Paramedic Long-term Care Program | Focus groups and semi structured interviews | Extended care Paramedic | Emergency Medical Service initiative: Extended Care Paramedic Programme | To identify insights gained and lessons learned during implementation and operation of this novel program. |
| North Dakota Center For Nursing | 2014 | USA | Policy Brief: Community Paramedic Pilot Study Recommendations | Policy | Community Paramedics | Community Paramedics via Emergency Medical Services | Policy recommendations to ensure that implementation of the Community Paramedicine Program will result in every patient receiving safe, quality care through the coordinated effort of all health care providers |
| O'Meara et al | 2014 | Canada | Community paramedicine: higher education as an enabling factor | Observational: Ethnographic case study using focus group and interviews (part of previous study - O'Meara et al 2016) | Community Paramedics | Emergency Medical Service initiative: Community Paramedic Programme | To describe a Canadian community paramedic model and to identify enablers related to successful implementation. |
| Agarwal et al | 2015 | Canada | Development of a community health and wellness pilot in a subsidised seniors’ apartment building in Hamilton, Ontario: Community Health Awareness Program delivered by Emergency Medical Services (CHAP-EMS) | Feasibility study | ‘Accommodated' paramedics who are unable to fulfil traditional paramedic duties. Patient population were elderly in subsidised housing with high frequency use of Emergency Medical Services | Emergency Medical Service initiative (The Community Health Assessment Program) | To measure the feasibility and challenges of implementing a Community Health Assessment Program through Emergency Medical Service (CHAP-EMS). |
| Clay and Stern | 2015 | UK | Making Time in General Practice | Case Study | Paramedic or Emergency Care Practitioner | General Practice: Home Visiting | Report for workforce recommendations to optimise general practice workforce. |
| Cope | 2015 | UK | How employing a paramedic solved our recruitment problem | Case Study | Paramedic | General Practice | Overview of how employing a paramedic assisted in filling a gap in the workforce. |
| Glendenning and Jones | 2015 | USA | New Hanover Community Paramedicine Success Story | News Article | Community Paramedics | Mobile Integrated Health Service (within Emergency Medical Services) | Outline of the beneficial role of the community paramedic within one healthcare region in |
| Heinelt et al | 2015 | Canada | Prehospital Identification of Underlying Coronary Artery Disease by Community Paramedics | Case Report (Part of Expanding Paramedicine in the Community (EPIC) study) | Community Paramedic | Emergency Medical Service initiative: Community Paramedicine at Home programme (home visiting) | To investigate the use of community paramedics in chronic disease management. |
| Kusel and Savino | 2015 | USA | Boots on the Ground: Alameda County, California Community Paramedics Curb Hospital Readmissions and Non-emergent 9-1-1 Use | News article | Community Paramedics | Emergency Medical Services | Description of a mobile integrated healthcare and community paramedicine (MIH-CP) implemented in one county within California. |
| NHS England | 2015 | UK | Improving Access with an Urgent Care Team at Beacon Medical Group, South | Case Study | Advanced Paramedic | Urgent Care Team within Medical Group | Overview of how employing a paramedic assisted in filling a gap in the workforce. |
| O'Meara et al | 2015 | Canada | Integrating a community paramedicine program with local health, aged care and social services: An observational ethnographic study | Observational: Ethnographic case study using focus groups, direct observation and informal discussions (part of previous study - O'Meara et al 2016) | Community Paramedics; Patients; other healthcare professions | Emergency Medical Service initiative: Community Paramedic Programme | To identify and describe the nature of the relationship between public engagement and the integration of community paramedicine with local health, aged care and social services |
| Turner et al | 2015 | UK | What evidence is there on the effectiveness of different models of delivering urgent care? A rapid review | Rapid evidence review | Paramedics employed in extended care roles | Ambulance services; community roles; emergency departments. | To assess the nature and quality of the existing evidence base on delivery of emergency and urgent care services and identify gaps that require further  primary research or evidence synthesis. |
| Abrashkin et al | 2016 | USA | Providing Acute Care at Home: Community Paramedics Enhance an Advanced Illness Management Program—Preliminary Data | Observational Study | Community Paramedics | Advanced Illness Management (AIM) programme run by Emergency Medical Services | To explore the feasibility of in-home evaluation and treatment of acute illnesses by paramedics within an Advanced Illness Management (AIM) program. |
| Beacon Medical Group | 2016 | UK | Meet our Advanced Paramedic Practitioner | Newsletter | Advanced Paramedic Practitioner | General Practice | Introduction of the new role of Advanced Paramedic Practitioner to medical group. |
| Brydes et al | 2016 | Canada | The CHAP-EMS health promotion program: a qualitative study on participants’ views of the role of paramedics | Participant observation and semi-structured interviews | Advanced Care Paramedics; Community Paramedics; Patients | Emergency Medical Service initiative (The Community Health Assessment Program) | To examine participants’ perceptions of paramedics providing a community paramedicine program, named the Community Health Assessment Program through Emergency Medical Services (CHAP-EMS). |
| Butcher, l | 2016 | USA | Tapping the Potential of Community Paramedicine | News article | Community Paramedics | Community Paramedics via Emergency Medical Services | Interview-style news article with the Director of California Emergency Medical Services Authority. |
| Colver et al | 2016 | UK | Paramedic Practitioner: A Survey of Scope of Practice and Development Requirements | Online cross-sectional questionnaire | Paramedic Practitioner | Scottish Ambulance Service | To describe paramedic practitioners’ views on their scope of practice and identify areas for improvement. |
| Dalgarno, D | 2016 | Canada | Community Paramedic in Home Blood Transfusions | Conference presentation | Community paramedics give blood transfusions to frail patients with mobility issues | Emergency Medical Service initiative: Community Paramedic Programme | To show that transfusions can be done safely in the home by community paramedics, to improve access to this care, and to improve the patient experience. |
| Haebler and Montera | 2016 | USA | Coming to a location near you: Community paramedics | Opinion Piece from American Nurse Association | Community Paramedics | Community Paramedics via Emergency Medical Services | Commentary and opinion on the development of the community paramedic role within the USA. |
| Imison, Castle-Clarke and Watson | 2016 | UK | Reshaping the workforce to deliver the care patients need | Report | Paramedic Practitioner; primary care practitioner’ | General Practice | To give practice guidance to reshape the general practice workforce. |
| Kizer | 2016 | USA | Community Paramedicine Builds a Much Needed Bridge to Quality Care | News Article | Community Paramedics | Community Paramedics via Emergency Medical Services | Introduction of the role of the community paramedic. |
| Long et al | 2016 | Australia | What’s in a name? The confusion in nomenclature of low-acuity specialist roles in paramedicine | Commentary | Any low-acuity specialist roles in paramedicine | Australia, Canada, UK and USA | Outline that consistent nomenclature for low-acuity paramedic roles in fundamental for professionalisation of the paramedic profession. |
| Martin et al | 2016 | Canada | Consumer perspectives of a community paramedicine program in rural Ontario | Observational: Ethnographic approach with informal discussions, semi-structured interviews and direct observation | Community Paramedics | Emergency Medical Service initiative: Community Paramedic Programme in Ontario | To evaluate a community paramedicine program in rural Ontario, Canada, through the perceptions and experiences of consumers. |
| Montera | 2016 | USA | The Community Paramedic’s Role in Treating Mental and Behavioural Health Patient | Opinion Piece | Community Paramedics | Emergency Medical Service | Outlines the use of community paramedics to work with patients with mental illness. |
| NHS England | 2016 | UK | Improving access: Paramedic practitioner service in the South Kent Coast | Case Study: Ambulance service provides paramedics to General Practice | Paramedic Practitioner | Employed by South East Coast Ambulance Service to undertake home visits on behalf of General Practice | Overview of how employing a paramedic assisted in the provision of a 7-day primary care service. |
| NHS England | 2016 | UK | Reducing pressure in general practice: Practice based paramedics, S Kent Coast | Case Study | Paramedic Practitioners | Employed by South East Coast Ambulance Service to undertake home visits on behalf of General Practice | Overview of the introduction of the paramedic practitioner role to provide a 7-day home visiting service. |
| NHS Salford Clinical Commissioning Group | 2016 | UK | Primary Care (General Practice) Workforce Development Strategy | Workforce Plan | Paramedics | Home visiting services | Sets out how Salford CCG will support and enable primary care providers to develop a multi-disciplinary workforce, in the right numbers with the appropriate knowledge, skills and values, to provide high quality primary care for the residents of Salford. |
| Northumberland LMC | 2016 | UK | A Primary Care Strategy for Northumberland 2016-2020 | Strategy | Community Paramedic; Advanced Paramedic | General Practice; Home visiting services | A vision for a vibrant and sustainable future for primary care. |
| O'Meara et al | 2016 | Canada | Community paramedicine model of care: an observational, ethnographic case study | Ethnography: direct observation, informal discussions, interviews and focus groups | Community Paramedics; Patients; other Healthcare Professions | Emergency Medical Service initiative: Community Paramedic Programme | To identify and analyse how community paramedics create and maintain new role boundaries and identities in terms of flexibility and permeability and through this develop and frame a coherent community paramedicine model of care that distinguish the model from other innovations in paramedic service delivery. |
| Patterson et al | 2016 | USA | What Is the Potential of Community Paramedicine to Fill Rural Health Care Gaps? | Structured interviews with programme leaders | Community Paramedics | Community Paramedics via Emergency Medical Services | ﻿To examine the goals, activities, and outcomes of 31 rural-serving community paramedicine programs through structured interviews of program leaders and document review. |
| Pennel, C et al | 2016 | USA | Emergency Medical Service- based Care Coordination for Three Rural Communities | Comparative case study approach: Interview and focus groups | Rural Community Paramedics working in care coordination roles | Emergency Medical Services | To report on the innovative care coordination programmes using local Emergency Medical Services to address the health and social care needs of rural populations in one American State. |
| Wilcox | 2016 | USA | Community Paramedicine in a Rural Setting | News article | Community Paramedics | Rural areas with predominant health inequalities | Description of provision of community paramedics via Emergency Medical Services in rural areas with predominant health inequalities. |
| Agarwal et al | 2017 | Canada | Effectiveness of a community paramedic led health assessment and education initiative in a seniors’ residence building: the Community Health Assessment Program through Emergency Medical Services (CHAP-EMS) | Prospective pre-post approach intervention study | ‘Accommodated' paramedics who are unable to fulfil traditional paramedic duties. Patient population were elderly in subsidised housing with high frequency use of Emergency Medical Services | Emergency Medical Service initiative (The Community Health Assessment Program) | To examine the effectiveness of the Community Health Assessment Program through Emergency Medical Service (CHAP-EMS) in reducing blood pressure, diabetes risk, and EMS calls. |
| Bowles et al | 2017 | Canada | Four Dimensions of Paramedic Practice in Canada | Semi structured interviews | 17 current or former paramedics in clinical, provincial or national leadership positions | Emergency Medical Service initiative: Community Paramedic Programme | To explore current conceptions of the terms, roles, boundaries and future directions of Canadian paramedic practice, and sought to identify key issues and problems facing stakeholders in Canadian paramedic education. |
| Brown | 2017 | UK | A day in the life of a paramedic advanced clinical practitioner in primary care | Case study | Advanced Clinical Practitioner (Paramedic) | Primary Care | To illustrate the range and complexity of the role of paramedics based in primary care, and how this role is developing beyond the traditional scope of paramedic practice. |
| Eaton | 2017 | UK | Taking healthcare to the community: the evolving role of paramedics | Opinion piece | Specialist Paramedic | First Aid Unit (staffed by South Central Ambulance Service) | Description of static first aid unit in North Oxfordshire and provision of services to the community. |
| Flomenbaum | 2017 | USA | Back to the Future, Part 2: Community Paramedicine | Opinion Piece | Community Paramedics | Community Paramedics via Emergency Medical Services | Commentary and opinion on the development of the community paramedic role within the USA. |
| Guo et al | 2017 | Canada | Community paramedicine: Program characteristics and evaluation. | Report including a systematic literature review | Community Paramedic Programmes. | Evaluates published literature in Australia, Canada, UK and USA | ﻿To provide a summary of information regarding currently existing community paramedicine programs. |
| Long | 2017 | Australia | Out of the silo- A qualitative study of paramedic transition to a specialist role in community paramedicine | Doctoral Thesis: Qualitative interviews | Community Paramedic | Emergency Medical Services employing community paramedics in Australia and Canada | To understand how qualified paramedics transition to specialist roles in community paramedicine. |
| McCarthy et al | 2017 | USA | Impact of Community Paramedic Program on Health Service Utilization | Retrospective review | Community Paramedics | Home visiting service provided by an urban Emergency Medical Service | To assess the impact of the community paramedic program on the number of Emergency Department visits and hospital admissions among patients enrolled in a community paramedic program. |
| Mogridge | 2017 | UK | Paramedic Practitioners in Primary Care | Conference Presentation | Paramedic Practitioners | Home visiting services | Overview of pilot scheme where paramedics employed by ambulance service undertake home visiting services. |
| NHS Ashford Clinical Commissioning Group; NHS Canterbury and Coastal Clinical Commissioning Group | 2017 | UK | CCG Operating Plan 2017-2019 | Workforce Plan | Paramedic Practitioners | General Practice | Outline of the planned implementation of local care models. |
| NHS England North | 2017 | UK | Expanding general practice workforce will transform patient care | News article | Community Specialist Practitioner (Paramedic) | General Practice | Overview of operationalisation of the General Practice Forward View in Cheshire and Merseyside. |
| NHS Thurrock CCG | 2017 | UK | First Primary Care Paramedic Arrives in Thurrock | News article | Emergency Care Practitioner Paramedic / Primary Care Paramedic | General Practice | Introduction of the role of paramedic to GP surgeries in clinical commissioning group. |
| RSM | 2017 | UK | Better Local Care Hampshire Multispecialty Community Provider Vanguard Deep Dive Evaluation Report: Paramedic Home Visiting Service | Interviews with clinical staff; survey of GPs; patient survey | Specialist Paramedic | Delegated home visiting services undertaken by South Central Ambulance Service | To evaluate Paramedic Home Visiting Services (PHVS). |
| Ruest et al | 2017 | Canada | Community Health Evaluations Completed using Paramedic Service (CHECUPS): Design and Implementation of a New Community-Based Health Program | Evaluation | Community Paramedics | Emergency Medical Service initiative: Community Paramedic Programme in Ontario | To review the “Community Health Evaluations Completed Using Paramedic Service” (CHECUPS) Program, a response to the Government of Ontario’s desire to evaluate how CP can contribute to the health care system in the Province of Ontario. |
| Sawyer and Coburn | 2017 | USA | Community Paramedicine: 911 Alternative Destinations Are a Patient Safety Issue | Opinion Piece | Community Paramedics | Community Paramedics via Emergency Medical Services | To outline concerns regarding the implementation of the Community Paramedic model of care. |
| Singh et al | 2017 | USA | ﻿Qualitative Evaluation of the Coach Training within a Community Paramedicine Care Transitions Intervention | Semi-structured interviews with community paramedics | Community paramedics | Community Paramedics via Emergency Medical Services | ﻿To define community paramedics' perceptions regarding their training needs to serve as care transition intervention coaches supporting the emergency department-to-home transition. |
| Spence | 2017 | UK | Good medicine — the GP paramedic | Opinion piece | GP Paramedic | Primary Care | Opinion on the positive contribution paramedics can make to general practice |
| Ulintz, A | 2017 | USA | Community Paramedicine in a Primary Care Group Practice | Case Study in conference presentation | Community Paramedics | Home visiting service | Description of Community Paramedics replacing physicians in a home visiting service. |
| Wessex Academic Health Science Network | 2017 | UK | The Wessex Primary Care Project | Workforce Plan | Paramedic | General Practice | Describe a range of initiatives that general practice can use to meet population and system demands. |
| Wood et al | 2017 | Canada | The Economic Value of Community Paramedicine Programs | Randomized controlled trial CT and economic analysis | Community Paramedics | Emergency Medical Service initiative: Community Paramedic Programme | To determine whether Community Paramedicine services could reduce hospital service utilization for high-frequency chronic condition clients and establish whether Community Paramedicine could influence self-perceived quality of life. |
| Abrashkin et al | 2018 | USA | Hospice at Home: Paramedics as Part of the Team | Case Study in conference presentation | Community Paramedics | Hospice Centre Network (alternative to Emergency Medical Services) | Description of the introduction of community paramedics to a hospice programme providing emergency visits as an alternative to 911. |
| Agarwal et al | 2018 | Canada | Evaluation of a community paramedicine health promotion and lifestyle risk assessment program for older adults who live in social housing: a cluster randomized trial | Open-label pragmatic cluster-randomized controlled trial with parallel intervention and control groups | Community Paramedics: ‘Accommodated' paramedics who are unable to fulfil traditional paramedic duties. Patient population were elderly in subsidised housing with high frequency use of Emergency Medical Services | Emergency Medical Service initiative: Community Paramedic Programme | To determine if a community paramedicine program - in which paramedics provide health care services outside of the traditional emergency response - reduced the number of ambulance calls to subsidized housing for older adults. |
| Barird et al | 2018 | UK | Innovative models of general practice | Report and Case Study | Paramedic | General Practice | To recommend a place-based approach to health and care, rooted in communities. |
| Bennett et al | 2018 | USA | Community Paramedicine Applied in a Rural Community | Pre/post-test with a comparison group study design | Community Paramedics | Emergency Medical Services | To determine if the community paramedic program reduced emergency department visits in one American county while improving patient outcomes. |
| Burns | 2018 | UK | GP perspectives of paramedic referrals to urgent and primary care | Interview and thematic analysis | Paramedic | Ambulance services | To gain more understanding  about how paramedics relate to urgent and primary care |
| Central LHIN Community Paramedicine Working Group | 2018 | Canada | Recommendations for Central LHIN Community Paramedicine | Report | Community Paramedic (CP) | Emergency Medical Service | To advance a consistent CP program across the Central LHIN to support improving the health of vulnerable and complex patients. |
| Chellappa et al | 2018 | USA | Supporting the on-call primary care physician with community paramedicine | Case Study | Community Paramedics (oversight from primary care physicians) | Home visiting service provided by primary care service | ﻿To describe the development of a community paramedicine programme that supports on-call primary care providers managing a high-risk patient population with the goal of reducing avoidable emergency department referrals. |
| Clarke | 2018 | UK | A Guide for General Practice Employing a Paramedic | Guideline | Paramedic; Specialist Paramedic; Advanced Paramedic | General Practice; Home visiting services | Outline of role descriptors, salary and clinical capabilities for paramedics working in general practice. |
| Commissioner of Community and Health Services | 2018 | Canada | Discontinuation of the Expanding Paramedicine in the Community Program | Report | Community Paramedic | Emergency Medical Service initiative: Community Paramedicine at Home programme (home visiting) | To provide information about discontinuing the Expanding Paramedicine in the Community (EPIC) program due to a withdrawal of funding from the Central Local Health Integration Network. |
| Dainty et al | 2018 | Canada | Home Visit-Based Community Paramedicine and Its Potential Role in Improving Patient-Cantered Primary Care: A Grounded Theory Study and Framework | Interviews and ethnography | Community Paramedics; Patients with chronic conditions. | Emergency Medical Service initiative: Community Paramedicine at Home programme (home visiting) | To study the patient perspective and valuation of this type of program to understand its potential value for primary care innovation in the future. |
| Graham | 2018 | Canada | Community Paramedic: A Practitioner's Perspective | Opinion piece | Advanced Care Paramedics; Community Paramedics | Emergency Medical Service initiative: Community Paramedic Programme | Opinion of the role, education and call types that Advanced Care Paramedics/Community Paramedics attend. |
| Health Education England | 2018 | UK | Community multi-disciplinary team rotation | News article | Specialist and Advanced Paramedics | Rotational between ambulance service and general practice | Outline of the inclusion of specialist and advanced paramedics within a multidisciplinary team. |
| Health Education England | 2018 | UK | Primary care rotation | Case study | Paramedic | General Practice | Outline of role of paramedic in GP surgeries, including patient story. |
| Huang et al | 2018 | USA | Development of sustainable community paramedicine programmes: a case study in Pennsylvania | Case Study with four structured interviews | Community Paramedics | Community Paramedics via Emergency Medical Services | ﻿To explore the strategies used by active community paramedic programmes and investigate their operational status, community demographics, financial models and challenges for programme development. |
| Leyenaar et al | 2018 | Canada | A scoping study and qualitative assessment of care planning and case management in community paramedicine | Scoping Review | Community Paramedics | Emergency Medical Service initiative: Community Paramedic Programme | To contribute to paramedic practice by examining broad areas of care planning in CP, identifying gaps in the evidence, clarifying key concepts, and reporting on the types of evidence that address and inform practice. |
| Lezzoni et al | 2018 | USA | Early Experiences with the Acute Community Care Program in Eastern Massachusetts | Observational Study | Paramedics | Acute Community Care Program (ACCP) provided within an Emergency Medical Service | To examine visits and post-visit service use, deaths, on-scene activities, and self-reported patient satisfaction of the Acute Community Care Program (ACCP) to provide insight into patient outcomes during the programmes’ first 24 months. |
| Lincolnshire East Clinical Commissioning Group | 2018 | UK | Specialist paramedics to support patients in care homes | News article | Specialist Paramedics | Specialist Paramedics from East Midlands Ambulance Service undertake care home visits on behalf of General Practice | Outline of scheme where paramedics undertake care home visits on behalf of General practice. |
| Moule et al | 2018 | UK | Preparing non-medical clinicians to deliver GP out-of-hours services: lessons learned from an innovative approach | Pre and post intervention questionnaires | Paramedics | Out-of-hours service | To present the development and evaluation of one programme delivered in 2017 of paramedics seeking to work in OOHs services |
| Murray | 2018 | UK | How do advanced paramedics experience and manage uncertainty in primary care organisations? | Master's Thesis: Literature review | Advanced Paramedics (APs) | Primary Care | Seeks to identify how APs are engaging with uncertainty within their practice in primary care. |
| NHS England | 2018 | UK | Wokingham paramedic home visiting model | Case Study | Paramedic Practitioners | Home visits on behalf of General Practice | Overview of primary care lead integrated paramedic home visiting model. |
| NHS Leeds Clinical Commissioning Group | 2018 | UK | Innovative GP service shortlisted for national award | News article | Specialist Paramedic | Rotational model between Yorkshire Ambulance Service, home visiting services and General Practice | Outline of workforce project that was shortlisted for a Health Service Journal Value Aware in the workforce efficiency category. |
| NHS Scotland | 2018 | UK | National Health and Social Care Workforce Plan: Part 3 – Improving workforce planning for primary care in Scotland | Workforce Plan | Paramedics and Advanced Paramedics | Primary Care | Sets out recommendations and the next steps that will improve primary care workforce planning in Scotland. |
| NHS Wales | 2018 | UK | A Planned Primary Care Workforce for Wales | Report and Case Study | Advanced Paramedic Practitioner | Primary Care; Paramedics in Ambulance Service | To describe the direction required for employed and contracted staff by putting in place actions to secure, manage and support a sustainable primary care workforce shaped by local population needs and by prudent healthcare principles. |
| North West Ambulance Service NHS Trust | 2018 | UK | Community Specialist Paramedic, Ivan Scrase | Case Study | Community Specialist Paramedic | Community Care | Week in the life of narrative of paramedic working in community care. |
| O'Meara et al | 2018 | Australia; USA; Canada | Frontier and remote paramedicine practitioner models | Narrative review | Extended Care Paramedic; Community Paramedic | Inclusion of published literature in Australia, Canada, UK and USA | ﻿To review paramedic models of service delivery, with an emphasis on models that have the potential to improve the health and wellbeing of frontier and remote populations. |
| Overberger et al | 2018 | USA | Community Paramedicine Interventions to Reduce Emergency Department Visits and Rehospitalizations | Retrospective analysis | Community Paramedics | Home visiting service provided by an urban Emergency Medical Services | To examine the effectiveness of a Community Paramedicine programme for patients discharged from an inpatient setting in reducing the rate of unplanned return visits and increasing the rate of planned follow-up visits. |
| Pearce, Cody and White | 2018 | USA | EMS-Based Urgent Care in the Ramah (N.M.) Navajo Reservation | Opinion Piece (Case Study) | Community Paramedics | Static programme via Emergency Medical Services within the Navajo Nation (American Indian Reservation) | Use of community paramedics to provide care to American Indians. |
| Shah et al | 2018 | USA | Improving the ED-to-Home Transition: The Community Paramedic–Delivered Care Transitions Intervention— Preliminary Findings | Single-blind randomized controlled trial | Community paramedics | Community Paramedics via Emergency Medical Services | ﻿To describe a novel model of care that uses community-based paramedics to deliver a modified version of the evidence-based hospital-to-home Care Transitions Intervention to a new context: the emergency department-to-home transition. |
| Sibley et al | 2018 | UK | Independent evaluation of the North East Hampshire and Farnham Vanguard Fleet Rapid Home Visiting Service | Evaluation - interviews and survey | Paramedic Practitioners | Home visiting services | Independent evaluation of the impact of running multiple interventions, delivered at three levels (individual, practice and system), on seven GP Retention Intensive Support Sites. |
| Turner and Williams | 2018 | UK | An Evaluation of early stage development of rotating paramedic model pilot sites Final Report | Evaluation: face to face and telephone interviews; qualitative data analysis. | Specialist Paramedics (SPs); Advanced Paramedics (APs) | SPs and APs across four UK sites (South Central; South Hardwick; East Lincolnshire; Newcastle) rotate between primary care (Home visiting services and general practice) and ambulance service (999 response and work within ambulance control) | This report, commissioned by Health Education England, aims to evaluate the development of a rotating paramedic model of care delivery designed to address both the career aspirations of specialist paramedics and the combined workforce issues in ambulance services and primary care so that all, not just some, of the healthcare sectors can benefit. |
| Wickware | 2018 | UK | Paramedics to resume GP home visits under new rota system | Case Study | Paramedic Practitioners | Paramedic Practitioners employed by South East Coast Ambulance Service undertaking home visits on behalf of general practices in Sussex | Description of a rotational model where paramedic practitioners are employed by the ambulance service and work every 8 weeks undertaking home visits for local general practices. |
| Agarwal et al | 2019 | Canada | Rationale and methods of an Evaluation of the Effectiveness of the Community Paramedicine at Home (CP@home) program for frequent users of emergency medical services in multiple Ontario regions: a study protocol for a randomized controlled trial | Protocol: An open-label, pragmatic, randomized controlled trial with parallel intervention and control groups will be conducted in four paramedic services in Ontario | Community Paramedic | Emergency Medical Service initiative: Community Paramedicine at Home programme (home visiting) | To evaluate the impact of a community paramedicine home-visit intervention with frequent users on reducing ambulance calls, hospital visits, and admissions. |
| Agarwal et al | 2019 | Canada | Reducing 9-1-1 Emergency Medical Service Calls by Implementing A Community Paramedicine Program For Vulnerable Older Adults In Public Housing In Canada: A Multi-Site Cluster Randomized Controlled Trial | Open label, pragmatic, cluster-randomized controlled trial | Community Paramedics | Emergency Medical Service initiative: Community Paramedic Programme | To evaluate the change in mean EMS calls at the building level, comparing intervention and control buildings, across multiple community sites. |
| Booker and Voss | 2019 | UK | Models of Paramedic Involvement in General Practice | Editorial | Paramedic | General Practice | Commentary describing the challenges, expectations and support needed as paramedics move into primary care roles. |
| Burgess, L | 2019 | UK | Primary Care in Scotland | Report | Paramedic | Primary Care | Outlines how primary care operates in Scotland. |
| Cameron and Carter | 2019 | Canada | Community paramedicine: A patch, or a real system improvement? | Commentary | Community paramedics | Canada-informed, with population in Emergency Medical Service employment | To describe the roles and opportunities open to paramedics. |
| Chan et al | 2019 | Canada | Community paramedicine: A systematic review of program descriptions and training | Systematic Review | Community Paramedic | Community Paramedic Programmes across Canada | To identify the types of community paramedicine programs and the training for each program. |
| Fisher et al | 2019 | UK | Briefing: Understanding primary care networks | Report | First contact community paramedics | Primary Care | To examine the rationale for networks and explore the relevant evidence for the future of primary care networks. |
| Gregg et al | 2019 | USA | Systematic Review of Community Paramedicine and EMS Mobile Integrated Health Care Interventions in the United States | Systematic Review | Mobile Integrated Health care programmes and Community Paramedicine programmes | Emergency Medical Services | ﻿To describe the outcomes from community paramedicine and mobile integrated health care interventions on controlling health care costs while improving population health and both provider and patient satisfaction. |
| Health Education England | 2019 | UK | Paramedic Specialist in Primary and Urgent Care Core Capabilities Framework | Framework | Specialist Paramedic | Primary and Urgent Care | Description of the core capabilities for paramedics to work in primary care. |
| Jones et al | 2019 | UK | ARRIVE: Ambulance paramedics Responding to urgent patient Requests In general practice for home Visits - Evaluation development | Abstract from oral conference presentation | Paramedics | General Practice: Home Visiting | To describe the evidence base, theoretical underpinning and current initiatives; and determine the feasibility of undertaking a definitive evaluation of PPC in order to produce generalisable evidence to inform policy and practice. |
| Leyenaar et al | 2019 | Canada | Examining consensus for a standardised patient assessment in community paramedicine home visits: a RAND/UCLA-modified Delphi Study | Delphi study with 13 purposively selected national experts | Community Paramedics | Emergency Medical Service home visit initiative: Community Paramedic Programme | To investigate the level of consensus that could be found by a panel of experts regarding appropriate health, social and environmental domains that should be assessed in community paramedicine home visit programme. |
| Leyenaar et al | 2019 | Canada | What do community paramedics assess? An environmental scan and content analysis of patient assessment in community paramedicine | Environmental scan and content analysis | Community Paramedics | Emergency Medical Service home visit initiative: Community Paramedic Programme | To summarize the content of assessment instruments and describe the state of current practice in community paramedicine home visit programs. |
| Malmsbury Medical Partnership Patients Participation Group | 2019 | UK | Minutes of the meeting held on Monday December 9th 2019 at 19.00hrs | Meeting minutes | Paramedic | General Practice: Home Visiting | Introduction of a paramedic working in General Practice, undertaking home visits. |
| Martin and O'Meara | 2019 | Canada/USA | Perspectives from the frontline of two North American community paramedicine programs: an observational, ethnographic study | Ethnography | Community Paramedics | Two independent Emergency Medical Service provided community paramedic programmes in North America: Ontario (Canada) and Colorado (USA) | To identify the motivations, job satisfaction and challenges of community paramedics pioneering two independent programs in rural North America from their perspectives and those of their managers. |
| Mid and South Essex Health and Care Partnership | 2019 | UK | Paramedics in General Practice (sometimes called Emergency Care Practitioners ECPs) | Newsletter | Primary Care Emergency Care Practitioners | General Practice | Factsheet introducing role of paramedics in general practice. |
| NHS Castle Point and Rochford Clinical Commissioning Group and NHS Southend Clinical Commissioning Group | 2019 | UK | Paramedics help ensure you get the right care this Winter | News article | Paramedic Home Visiting Clinician | General Practice: Home Visiting | Outline of the paramedic role to help general practices meet demands. |
| NHS England | 2019 | UK | GP practices free up 3,000 extra patient appointments through Primary Care Network | News article | Paramedic | General Practice | Outline of changes introduced as part of a primary care network. |
| NHS England | 2019 | UK | GPs create 100,000 extra patient appointments through Primary Care Network model | News article | Paramedic | General Practice | Outline of changes introduced as part of a primary care network. |
| Oxford Primary Care Commissioning Committee | 2019 | UK | Primary Care Workforce Strategy | Workforce Plan | Paramedics | General Practice; Home Visiting Services; Rotational through ambulance service and primary care | Workforce strategy to deliver primary care services within Oxfordshire. |
| Pang et al | 2019 | USA | Limited data to support improved outcomes after community paramedicine intervention: A systematic review | Systematic Review | Community Paramedics | Community paramedic programmes according to the USA national definition (papers from Australia, Canada, UK and USA) | ﻿To describe the evidence supporting community paramedicine practice. |
| Primary Care One | 2019 | UK | Advanced Paramedic Practitioner (APP) Practice | News article | Rotational Advanced Paramedic Practitioners (APPs) | Welsh Ambulance Service NHS Trust (WAST) APPs rotate through General Practice | Overview of the APP role within WAST |
| Proctor | 2019 | UK | Home visits from paramedic practitioners in general practice: patient perceptions | Semi-structured interviews with thematic analysis | Paramedic Practitioners | Home visiting (within General Practice) | To explore older patients' perceptions of having PPs, who work in GP surgeries, attend to them on a home visit in place of the GP. |
| Rasku et al | 2019 | Finland | The core components of Community Paramedicine – integrated care in primary care setting: a scoping review | Scoping review | Community Paramedics | Community Paramedics via Emergency Medical Services; Primary Care Settings; other non-emergency services. | ﻿To identify and describe the core components of community paramedicine models of healthcare delivery and identify research gaps for the further study. |
| Abrams et al | 2020 | UK | Delegating home visits in general practice: a realist review on the impact on GP workload and patient care | Realist review | Community paramedic (alongside other healthcare professionals) | Home visiting services | To explore how the process of delegating home visits works, for whom, and in what contexts. |
| Baird et al | 2020 | UK | How to build effective teams in general practice | Guide | Paramedic | General Practice: Home Visiting | To bring together insights from research, policy analysis and leadership practice. |
| Dixon | 2020 | UK | The developing role of the paramedic prescriber | Case study | Paramedic | General Practice | Description of how independent prescribing fits within role in general practice. |
| Healthcare Improvement Scotland | 2020 | UK | ﻿Hospital at Home: Guiding principles for service development | Report | Paramedic | General Practice in Scotland | Source of information and evidence of ‘hospital at home’ services |
| Jones | 2020 | UK | My first 3 months as a Rotational Advanced Paramedic Practitioner | Case Study | Advanced Paramedic Practitioner | General Practice; Rotational through Welsh Ambulance Service and primary care | To share experiences of the first three months of rotational role between the ambulance service and primary care. |
| London Ambulance Service NHS Trust | 2020 | UK | Our paramedics to help ease pressure on GP services this winter | News article | Paramedic Home Visiting Clinician | Rotational through London Ambulance Service and home visiting roles in general practice | To improve the care patients, receive and reduce pressures on GPs during winter. |
| Mid Essex Clinical Commissioning Group | 2020 | UK | Services available from your GP surgery | News article | Paramedic Practitioners | General Practice: Home Visiting | Introduction of a paramedic working in General Practice, undertaking home visits. |
| NHS England | 2020 | UK | South Coast Medical Group Primary Care Network: Supporting the demand on primary care | Case Study | Paramedic | General Practice: Home Visiting | Introduction of a paramedic working in General Practice, undertaking home visits. |
| NHS England | 2020 | UK | Suffolk Coast and Country Primary Care Collaboration | Case Study | Paramedic | General Practice: Home Visiting | Outline of how the introduction of two paramedics have reduced General Practitioner workload. |
| NHS England | 2020 | UK | The changes being made | Case Study | Paramedic | General Practice: (Rapid) Home Visiting | Implementation of a paramedic rapid home visiting service to free up GP time. |
| NHS Wiltshire | 2020 | UK | Case Study: Gareth Ward | Case Study | Specialist Paramedic | General Practice | Overview of the career journey of a paramedic working in general practice. |
| NHS Wiltshire | 2020 | UK | Case Study: Lili Ratcliffe | Case Study | Home Visiting Paramedic | General Practice: Home Visiting | Overview of the career journey and role of a home visiting paramedic employed by general practice. |
| Royal College of General Practitioners | 2020 | UK | Multidisciplinary Toolkit | Introductory Guide | Primary Care Paramedic | General Practice | To support practices exploring how to develop their clinical teams and create a way of working that is better able to meet their population needs for both urgent and routine primary care. |
| Royal College of General Practitioners | 2020 | UK | Fit for the Future: Workforce Roadmap | Report | Paramedic | General Practice | To set out a future vision for general practice to meet the challenges and opportunities of the next decade. |
| Schofield et al | 2020 | UK | Exploring how paramedics are deployed in general practice and the perceived benefits and drawbacks: a mixed method scoping study | Mixed methods: Literature review; survey; qualitative interviews. | Paramedics | General Practice | To understand how paramedics are deployed in general practice, and to investigate the theories and drivers that underpin this service development. |
| Thurman et al | 2020 | USA | A scoping review of community paramedicine: evidence and implications for interprofessional practice | Scoping Review | Community Paramedics | Community paramedic programmes in Australia, Canada, UK and USA | ﻿To understand the evidence base of community paramedic programmes in order to inform further evolution of this model of care. |
| Wagstaff and Mistry | 2020 | UK | The Integration of paramedics into primary care | Case Study | Paramedic Practitioner | One general practice surgery | Description of the work undertaken by the author as a paramedic practitioner working in general practice. |
| Watkins | 2020 | UK | Paramedic uses her skills to make a difference in the community | Case Study | Advanced Paramedic Practitioner | General Practice | Outline of the role of an Advanced Paramedic Practitioner working for a general practice surgery in Wales. |
| Health Education England | 2021 | UK | First Contact Practitioners and Advanced Practitioners in Primary Care: (Paramedic) A Roadmap to Practice | framework | First Contact Paramedic, Advanced Paramedic | Primary Care | To provide a roadmap of education for practice when moving into First Contact Practitioner (FCP) roles, and onward to Advanced Practice (AP) roles in Primary Care. |
| NHS England | 2021 | UK | Supporting General Practice in 2021/22 | Briefing report | Paramedic; Paramedic Practitioner; Advanced Paramedic | General Practice | To support the workforce |
|  |  |  |  |  |  |  |  |

| **Table 2b. Table of Job Advertisements** | | | | |
| --- | --- | --- | --- | --- |
| **Employer** | **Year** | **Job Title** | **Role Overview** | **Salary** |
| Blackmore Vale Partnership | 2016 | Primary Care Paramedic Practitioner | Home visiting services; First point of contact within the practice for patients presenting with undifferentiated, undiagnosed problem; Act as the emergency care lead for the practice; Develop and set up new patient services; Provide clinical leadership. | NA |
| Concordia Health | 2017 | Paramedic | Home visiting service (including palliative care); Post-discharge reviews; Minor illness service; Onsite support as non-medical prescriber; Attendance at clinical meetings. | *Competitive* |
| AT Medics Ltd | 2018 | Paramedic Practitioner /Primary Care Paramedic | The role will provide a specialist Paramedic resource working in collaboration with other members of the multidisciplinary team with a focus on home visits, telephone consultations and practice-based appointments; To assess, diagnose, treat, refer or signpost patients who contact the surgery with undifferentiated or undiagnosed condition relating to minor illness; Under supervision and guidance of a GP, work clinical sessions consisting of telephone appointments, face to face appointments and home visits; to provide assessment, diagnosis and treatment at first point of contact by attending to patients according to patients’ needs either at practice sites or patient’s homes/place of residence. | (£38,000 - £45,000 p.a) Negotiable with benefits commensurate with position |
| Valentine Health Partnership | 2018 | Paramedic Practitioner | Assessing, diagnosing, treating and discharging patients autonomously and in reviewing and assessing common long-term conditions. Training will be considered for someone who wants to make the transition from a traditional paramedic role into general practice. | £45,000 |
| Brunel Health Group Primary Care Network | 2019 | Paramedic Practitioner | Peripatetic Paramedic Practitioners, to undertake the home visiting service | £40-45k pro rata |
| Care UK | 2019 | Urgent Care Practitioner (Paramedic) | The clinical dimensions of the post encompass the assessment, diagnosis and treatment of patients presenting with minor illness. All clinical activities will be within the scope of practice of the post holder. | £28/hour |
| Coast and Country Collaboration | 2019 | Senior Paramedic / Emergency Care Practitioner | To practice autonomously as a lone practitioner without direct supervision as a Senior Paramedic/Emergency Care Practitioner across the Coast and Country Collaboration area. The role will involve the assessment, diagnosis, treatment or referral of patients presenting with undifferentiated and undiagnosed conditions. The post holder will work autonomously within their level of competency. | AfC Band 6-7 |
| DHU Urgent Care (LLR) | 2019 | Emergency Care Practitioner | The post holder will work autonomously, undertake clinical assessment, make referrals as required and give advice to patients across a wide spectrum of clinical conditions within the services provided by DHU Urgent Care (LLR). | £45,076 |
| Downend Health Group | 2019 | Paramedic in General Practice | First point within the practice for patients presenting with undifferentiated, undiagnosed problems, making use of history taking, physical examination, problem-solving and clinical decision-making, to establish a diagnosis and management plan; Provide assessment, treatment and diagnosis at point of first contact, by attending to patient’s in a variety of clinical or non-clinical settings according to patients’ or practice needs; Undertake home visits, in accordance with practice protocols; Make professionally autonomous decisions; hold a virtual case load; To provide holistic patient care within the unscheduled care setting from assessment of presenting condition through to diagnosis and initial treatment including the dispensing of medication within agreed clinical guidelines (PGDs) and protocols. | NA |
| GTD Healthcare | 2019 | Advanced Practitioner Paramedic | Portfolio working across a walk-in-centre service, home visiting services, telephone triage, ED settings and GP primary and urgent care. | £40-48k pro rata (Potential earning of £44,000 - 76,800 WTE Inclusive of enhancements/ incentives and unsocial hour payments) |
| Lister House Surgery | 2019 | Paramedic Practitioner | Working under GP supervision, this newly created role is intended to support GPs by providing general medical care through assessment, diagnosis, treatment and referral or signposting to other services, subject to scope of practice and levels of qualification. It is anticipated the Primary Care Practitioner (paramedic) role will encompass participation in patient telephone & face-to-face triage, holding clinics, home visits, minor ailments, coworking with an Advanced Nurse Practitioner and general support to the GP team, with the potential to expand and design according to demand and specialist skills/experience. | £33,222-£43,041 |
| NELFT NHS Foundation Trust | 2019 | Primary Care Paramedic Practitioner ECP | Manage the local population under supervision from a General Practitioner or autonomously depending on the scope of their role, and will be able to assess, diagnose, treat or refer patients within the scope of professional qualifications and skills and abilities in a General Practice Setting. | Band 7: £33,222-£43,041 plus HCAS 5% supplement (£1,000-£1,733) |
| Penrose Surgery | 2019 | Paramedic | Working with the triage team; Offer telephone and face-to-face same day appointments for acute cases; See patients with minor illness and minor injuries; Home visits and Holistic reviews; Help with long term conditions and other clinical target areas. | *Competitive* |
| Poole Central | 2019 | Paramedic Primary Care / Advanced Paramedic Practitioner | Experienced clinician, with an interest in supporting frail and complex patients in the community to enable patients to remain cared for at home. | Equiv. band 7 |
| Stourside Medical Practice | 2019 | Advanced Paramedic Practitioner | Following appropriate triage, patients will be booked for either a GP or Advanced Paramedic Practitioner appointment depending on the complexity of the issue. The triage process may be undertaken by another member of the team or by the Advanced Paramedic Practitioner. There is a requirement to see extra or emergency patients. | AfC Band 8a £42,414,000 to £49,969 pro rata |
| Tadley Medical Partnership | 2019 | Primary Care Paramedic | The post-holder is an experienced Advanced Clinical Practitioner, who will be acting as a Triage/Paramedic Practitioner within their professional boundaries. The post-holder will provide care for the presenting patient from initial history taking, clinical assessment, diagnosis, treatment and evaluation of their care. | From £37,000 |
| The Mission Practice | 2019 | Paramedic - Primary Care Practitioner | The post holder will provide clinical care to patients of the practice by way of surgeries, clinics, triage clinic, on-call duties, home visits and relevant administrative work together with such other duties as are required of the practice which are reasonably delegated by the Practice. | TBC (does not follow AfC) |
| Albion Place Medical Practice | 2020 | Paramedic | Responsible for face-to-face and telephone consultations with patients presenting with a wide range of illnesses. Treatment provided will be both in the surgery and in patients own homes. Provision of care for the presenting patient from initial history taking, clinical assessment, diagnosis, evaluation of care and treatment where appropriate. | Band 6: £26,171 - £37,267 per annum |
| Care UK | 2020 | Paramedic | Provide high quality emergency care and minor injury treatment to patients with both primary care and acute care needs in a prison setting - performing procedures such as suturing, IV access, haemorrhage control, wound care and cardiac & cerebral vascular accident intervention. The post-holder will play an active part in the assessment, planning, implementation and evaluation of the patients care, working as part of a team of primary care nurses within HMP Wayland | £19/hour |
| Care UK | 2020 | Paramedic | Provide high quality emergency care and minor injury treatment to patients with both primary care and acute care needs in a prison setting - performing procedures such as suturing, IV access, haemorrhage control, wound care and cardiac & cerebral vascular accident intervention. The post-holder will play an active part in the assessment, planning, implementation and evaluation of the patients care, working as part of a team of primary care nurses within HMP Wormwood Scrubs and respond to emergencies. | up to £44,000 FTE - DOE |
| Care UK | 2020 | Paramedic | Provide high quality emergency care and minor injury treatment to patients with both primary care and acute care needs in a prison setting - performing procedures such as suturing, IV access, haemorrhage control, wound care and cardiac & cerebral vascular accident intervention. The post-holder will play an active part in the assessment, planning, implementation and evaluation of the patients care, working as part of a team of primary care nurses within HMP Brixton and respond to emergencies. | £44,044 per annum DOE |
| Care UK | 2020 | Paramedic | Provide high quality emergency care and minor injury treatment to patients with both primary care and acute care needs in a prison setting - performing procedures such as suturing, IV access, haemorrhage control, wound care and cardiac & cerebral vascular accident intervention. The post-holder will play an active part in the assessment, planning, implementation and evaluation of the patients care, working as part of a team of primary care nurses within HMP UPI Doncaster and respond to emergencies. | up to £39,000 per annum. |
| Cranfield Surgery | 2020 | Advanced Paramedic Practitioner | To deliver a high standard of patient care as an Advanced Paramedic Practitioner in general practice, using advanced autonomous clinical skills, and a broad and in-depth knowledge of theoretical knowledge; To formulate a management plan, using the multi-disciplinary team if required to support diagnosis or next course of action; To diagnose and manage acute, chronic and urgent long term conditions, prescribe medication and be accountable for all clinical decisions within clinical competence; Instigate necessary invasive and non-invasive diagnostic tests or investigations and interpret findings/reports. | NA |
| DHU Healthcare CIC | 2020 | Paramedic Practitioner | Responsible for the clinical assessment, treatment and/or referral or giving advice as appropriate to both adults and children presenting with a wide spectrum of clinical conditions either within the Primary Care Centre or while undertaking a Home Visit. | £53,536.86 pro rata |
| East Coast Community Healthcare CIC | 2020 | Paramedic | An autonomous practitioner that is responsible for their own caseload and take a lead in the delivery of projects to improve service delivery, patient safety and quality of care with the aim of admission prevention to the acute hospital carrying out rapid holistic assessments in patients own homes, including physical, physiological and social aspects of need including palliative and end of life care patients. | £30,401 to £37,267 |
| Farnham Road Practice | 2020 | Visitation Paramedic & Emergency Practitioner | The post holder will be responsible for the assessment, diagnosis, treatment and referral of patients within their level of competence supported by agreed protocols following initial triage by a GP. Farnham Road Practice has a team of paramedics so there will be an opportunity to work alongside and learn from this established team. | *Competitive* |
| G4S | 2020 | Paramedic | To provide high quality emergency care and minor injury treatment to patients with both primary care and acute care needs in a prison setting by playing an active part in the assessment, planning, implementation and evaluation of the patients care. | £22 p/hr Mon-Fri and £25 p/hr for nights and weekends. |
| Highham Ferrers | 2020 | Paramedic | Provide services including telephone, face to face appointments and home visiting service to those who are unable to attend the surgery with the aim of supporting them to stay at home safely. | *Competitive* |
| Integrated Care Partnership | 2020 | Paramedic Practitioner | The Paramedic Practitioner will act autonomously, within their professional scope of practice, providing care for patients primarily on home visits, visits to care homes, and through face-to-face and telephone consultations. | Depending on Experience |
| ISSA Medical Group | 2020 | Advanced Paramedic Practitioner | To work within the community as an autonomous, accountable, Advanced Paramedic Practitioner, in the provision of a holistic approach for individuals including assessment, diagnosis and treatment, to deliver quality patient services; To assess, diagnose, treat, refer or signpost patients/service users who attend surgery with undifferentiated or undiagnosed condition relating to minor illness or minor injury; The post holder will use advanced clinical skills to provide education to service users, promoting self-care and empower them to make informed choices about their treatment. | Salary dependant on experience |
| Kingswood Surgery | 2020 | Primary Care Paramedic | To assess, diagnose, treat, refer or signpost patients/service users who attend surgery with undifferentiated or undiagnosed conditions. | *Competitive Salary Package* |
| Nottinghamshire Healthcare NHS Foundation Trust | 2020 | Senior Paramedic | To provide high quality emergency care and minor injury treatment to patients with both primary care and acute care needs in a prison setting by playing an active part in the assessment, planning, implementation and evaluation of the patients care. To provide basic leadership to junior staff by maintaining professional standards ensuring adherence to all relevant policies and procedures to ensure the delivery of high-quality care. To provide support and supervision for junior qualified and unqualified staff by helping the line manager with staff development and by providing a first point of contact for patients when on duty. | £30,401- £37,267 per annum |
| Omnes Healthcare | 2020 | Paramedic Practitioner based in General Practice | Home visiting service (including palliative care); Post-discharge reviews; Minor illness service; Onsite support as non-medical prescriber; Attendance at clinical meetings. | *Competitive* |
| OnePrimaryCare | 2020 | Paramedic Practitioner | The post holder will be part of wider multi-disciplinary team within Primary Care and will work within their personal scope of practice to deliver excellent care – supporting the practice values. The Paramedic Practitioner will predominantly support the delivery of acute on the day care. This may involve elements of telephone and face to face triage, acute on the day clinics and home visits. | Up to £35,000 pro rata (depending on experience) |
| Portsdown Group Practice | 2020 | Paramedic Practitioner | Role will be varied and wide-ranging, which can include telephone triage, face-to-face same-day consultations and home visits, supported by an experienced team of dedicated clinicians. The Paramedic Practitioner at Portsdown Group Practice will be an experienced practitioner who, acting within their professional boundaries, will provide care for patients presenting at the practice from initial history taking, clinical assessment, diagnosis, treatment and evaluation of care. They will demonstrate safe, clinical decision-making and expert care, including assessment and diagnostic skills, for patients within the general practice. | £42,750 - £48,500 |
| Princes Medical Centre | 2020 | Paramedic | An experienced Paramedic Practitioner who has experience in prescribing for minor illness, minor injuries and long-term conditions. The post-holder will demonstrate safe, clinical decision-making and expert care for patients within general practice. The post-holder will work collaboratively with the multi-disciplinary general practice team to meet the needs of patients and support the delivery of policy and procedures. | £44,606 to £50,819 pa |
| Southampton Primary Care Ltd | 2020 | Specialist Paramedic (Practitioner) | An experienced nurse practitioner/Specialist Paramedic acting within their professional boundaries will provide care for the presenting patient from initial history taking, clinical assessment, diagnosis, treatment and evaluation of their care. They will demonstrate safe, clinical decision-making and expert care for patients within the general practice. | £35/hour with prescribing qualification |
| Suffolk Primary Care | 2020 | Practice Paramedic | Act as a senior paramedic/emergency care practitioner, demonstrating advanced clinical competence and a knowledge base beyond those associated with conventional nursing roles; Undertake consultations in the surgery or the community as an autonomous practitioner and using own clinical judgement to diagnose and treat patients; Make direct referrals to primary, secondary and social services within locally agreed pathways, guidance and protocols; Ensure that patients receive high quality clinical care, delivered in a timely manner; Supply and administer medicines as indicated to address patient need, where necessary working to Patient Group Directions or with reference back to registered GP as necessary; Ensure complete and accurate documentation of each and every patient contact. | *Competitive* |
| The Light Surgery | 2020 | Paramedic Practitioner | The Paramedic Practitioner will predominantly support the delivery of acute, on the day care. This may involve elements of telephone and face to face triage, acute on the day clinics and home visits. | Up to £35,000 annual pro rata (depending on experience) |
| West Kent CCG | 2020 | Advanced Paramedic Practitioner | Autonomously assess, manage and treat patients presenting to the primary care service and work closely with General Practitioners to form a duty team each day. | £50,000 - £60,000 (pro rota) |
| Westway Surgery | 2020 | Primary Care Practitioner (Paramedic) | To work as an autonomous experienced practitioner, acting within professional boundaries to provide care for the presenting patient. Working as part of the duty team provide telephone triage and face to face consultations for acute and urgent presentations demonstrating safe, clinical decision-making and expert care for patients. | £38,890 - £44,503 pro rata (Agenda for Change Band 7 equivalent) |
| Essex Primary Care | 2021 | Paramedic Primary Care | To work within the community, as an autonomous, accountable, Specialist / Advanced Paramedic, in the provision of a holistic approach for individuals including assessment, management and treatment, to deliver high quality patient services; To assess, manage, treat, refer and/or signpost patients/service users who attend surgery with undifferentiated or undiagnosed condition relating to minor illness or minor injury; The post holder will use advanced clinical skills to provide education to service users, promoting selfcare and empower them to make informed choices about their treatment. | Depending on Experience |
| NHS England | 2021 | Example Job Description Paramedic | Work autonomously within the community at an advanced level of practice, using their enhanced clinical assessment and treatment skills, to provide first point of contact for patients presenting with undifferentiated, undiagnosed problems relating to minor illness or injury, abdominal pains, chest pains and headaches. They are health professionals who practice at an advanced level having the capability to make sound judgements in the absence of full information and to manage varying degrees of risk when there are complex, competing or ambiguous information or uncertainty. | AfC Band 6-7 |
|  |  |  |  |  |
