# Supplementary material for: Understanding the role of the paramedic in primary care: a realist review
Source: BMC Med. 2021 Jun 25;19:145. doi: 10.1186/s12916-021-02019-z (PMC8229679; doi:10.1186/s12916-021-02019-z)
Supplement: Supplementary file 3 — Additional file 3. Final Programme theory. [file 12916_2021_2019_MOESM3_ESM.docx]

**Abstract Category 1: Expectations of paramedics working in primary care**

***Patient Perspectives***

Patients want to know what paramedics do in their General Practice. When the paramedic role in General Practice is clear to patients (C) they understand how the role is appropriate in relation to their care needs (M), and so have increased confidence when they are treated by paramedics (O)

When a trusted source explains the role and value of seeing a paramedic (C), patients understand how the role is appropriate in relation to their care needs (M), and so are supportive of the introduction of these new roles (O) and have increased confidence when they are treated by paramedics (O)

When the care provided by paramedics meets the patients’ expectations (C), they will be more willing to be seen by a paramedic in the future (O) and more satisfied with the appointment outcome (O) because they are reassured with the level of care provided (M)

When a paramedic uses their longer appointment time to listen and understand a patient’s problem (C), patients are more willing to see them again (O), because they value this approach (M)

When paramedics have a therapeutic relationship with the patients they see (C), these patients are reassured with the level of care provided (M) and more willing to be seen by a paramedic in the future (O) and more satisfied with the appointment outcome (O)

When patients want to be seen by their usual GP (C), they do not wish to be seen by a paramedic (O) as this is not what they expect (M).

***GP Perspectives***

GPs do not regard paramedics as diagnosticians (O) and so employ them in assessment-only roles (C) as they consider the paramedic to be ‘out of depth’ in primary care (M)

When the employer does not consider the paramedic to have the skills and competencies relevant for their needs (C) employers may be less likely to employ paramedics (O) because they don't consider them to be useful (M)

When the paramedics can demonstrate that they can help to reduce the workload of GPs (C), because this is valued by GPs (M) paramedics continue to be actively recruited into primary care (O)

Paramedics are actively recruited into primary care (O) when gaps within the workforce exist (C) because they are perceived to be able to support general practice (M)

***Paramedic Perspectives***

Paramedics who perceive their role as a generalist (C) will look for opportunities for employment in primary care (O) because they believe they can enjoy and work in that environment (M)

***Contribution to Primary Care Teams***

When Emergency medical services use rotational models for paramedics to work in both the ambulance service and primary care (C) the needs of both organisations are more likely to be met (such as EMS workforce retention and increased workforce capacity in primary care) (O) because paramedic staff retention rates and satisfaction is higher (M).

When sufficient financial reimbursement is offered for paramedic roles in primary care (C) because paramedics are viewed as a credible addition to the team by employers (M) they are considered for recruitment into the practice workforce (O)

The existing skills and knowledge of paramedics is perceived by commissioners and stakeholders to correlate well into primary care (C) and so paramedics are actively recruited into primary care (O) because what they can offer is valued (M)

For whom: Rural workforces/teams

When paramedics provide access to healthcare which otherwise would not be available (C) they are considered a community asset (O) because what they do is highly valued by patients and service commissioners and providers (M)

**Abstract Category 2: Transition from EMS into primary care roles**

***Education***

Paramedics are able to transition into advanced practice roles (O) when they are supported by their employers in primary care (M) with clinical supervision (C) and access to formal education (C)

As paramedics transition from EMS to primary care roles (C), paramedics move away from their traditional scope of practice (O) because of the change in the conditions they have to manage (M)

***Supervision***

When paramedics are clinically supported in general practice (C), because they feel better supported (M) they will continue to advance and/or develop their capabilities and confidence within their role (O)

When paramedics are clinically supported in general practice (C), because they feel better supported (M), they have higher satisfaction with their role (O)

When paramedics have easy access to clinical supervision and advice (C), they have higher satisfaction with their role (O) because they feel better supported (M)

***Experience***

Paramedics at a junctional point within their career (C) value the opportunity to develop themselves (M) and so look for opportunities for employment in primary care (O)

Paramedics are pluripotential (C). Because of the breadth of issues with which they can deal (M) paramedics are considered a useful addition to the primary care team (O)

**Abstract Category 3: Role and Responsibilities**

***Working in a Team***

When the professional role boundaries of paramedics overlap with existing health care professionals in General Practice (C) there may be resistance of the paramedic role and responsibilities by these other health care professionals (O), because they feel threatened (O)

There is dysfunction to the employment of paramedics in primary care (O) when the role and responsibilities of the paramedic are unclear (C). When this occurs, paramedics are less likely to be empowered (M) and work within the full range of their capabilities (M)

When the paramedics capabilities have been demonstrated (C) they are viewed by practice staff as a credible addition to the team (M) and are accepted into the practice workforce (O)

When the boundaries of the paramedic scope of practice are understood by the practice team (C), paramedics are viewed as a credible addition to the team (M) and are accepted into the practice workforce (O)

***Interpersonal skills***

When paramedics demonstrate to the GP practice their awareness of their professional competencies (C), other health care professionals are more likely to accept the paramedic role in the practice (O), because they develop trust in their abilities (M)

When paramedics in a patient consultation display strong interpersonal skills and enthusiasm (C) patients are more likely to be satisfied (O) because patients use these to judge the quality of care they received (M)
